# Interactions between Social Structure, Demography, and Transmission Determine Disease Persistence in Primates

**DOI:** 10.1371/journal.pone.0076863

**Published:** 2013-10-18

**Authors:** Sadie J. Ryan, James H. Jones, Andrew P. Dobson

**Affiliations:** 1 Department of Environmental and Forest Biology, State University of New York College of Environmental Science and Forestry, Syracuse, New York, United States of America; 2 Center for Global Health and Translational Science, Department of Immunology and Microbiology, State University of New York Upstate Medical University, Syracuse, New York, United States of America; 3 Department of Agriculture, Engineering, and Science, School of Life Sciences, University of KwaZulu-Natal, Pietermaritzburg, South Africa; 4 Department of Anthropology, Stanford University, Stanford, California, United States of America; 5 Department of Ecology and Evolutionary Biology, Princeton University, Princeton, New Jersey, United States of America; 6 Santa Fe Institute, Santa Fe, New Mexico, United States of America; Arizona State University, United States of America

## Abstract

Catastrophic declines in African great ape populations due to disease outbreaks have been reported in recent years, yet we rarely hear of similar disease impacts for the more solitary Asian great apes, or for smaller primates. We used an age-structured model of different primate social systems to illustrate that interactions between social structure and demography create ‘dynamic constraints’ on the pathogens that can establish and persist in primate host species with different social systems. We showed that this varies by disease transmission mode. Sexually transmitted infections (STIs) require high rates of transmissibility to persist within a primate population. In particular, for a unimale social system, STIs require extremely high rates of transmissibility for persistence, and remain at extremely low prevalence in small primates, but this is less constrained in longer-lived, larger-bodied primates. In contrast, aerosol transmitted infections (ATIs) spread and persist at high prevalence in medium and large primates with moderate transmissibility;, establishment and persistence in small-bodied primates require higher relative rates of transmissibility. Intragroup contact structure – the social network - creates different constraints for different transmission modes, and our model underscores the importance of intragroup contacts on infection prior to intergroup movement in a structured population. When alpha males dominate sexual encounters, the resulting disease transmission dynamics differ from when social interactions are dominated by mother-infant grooming events, for example. This has important repercussions for pathogen spread across populations. Our framework reveals essential social and demographic characteristics of primates that predispose them to different disease risks that will be important for disease management and conservation planning for protected primate populations.

## Introduction

Recent catastrophic declines in African gorilla and chimpanzee populations have illustrated the impact that infectious disease can have on wild populations [1]–[4], yet we rarely hear of similar disease impacts to Asian great apes, or to smaller primates. In this paper, we developed an age-structured model framework for different primate species to examine how the interaction between social system and demography predisposes them to invasion by a potentially lethal pathogen that produces no immunity, such as a novel spillover pathogen like Ebola [3] or circulating human respiratory pathogens [1].

Primate species exhibit a wide range of variation in group and population structures; their life-history traits broadly correlate with social system type, with small-bodied primates typifying the monogamous and solitary social systems, and larger species such as chimpanzees (*Pan troglodytes*) having complex social interactions in larger groups [5]. There are important exceptions to this, such as the Asian great apes: orangutans (*Pongo sp.*) which are large, yet mostly solitary. From an epidemiological perspective, the combination of social contact structure and demographic rates confound the assumptions of well-mixed population models for disease establishment and initial epidemic outbreaks in a population [6]; the consequences of this have not until now been systematically explored from the comparative evolutionary perspective of how changes in social system between similar species modify epidemic potential.

We hypothesize that a combination of social structure and demography predispose some primates to greater disease impact, and that pathogen transmission mode is central to this. In particular, we hypothesize that large primates with complex group structures (social systems that involve more than a mating pair and their respective offspring, as in solitary and monogamous systems) are more likely to sustain pathogens with frequency dependent transmission, than will smaller primates with similar group structures, or large primates with simpler, less polygamous, social structures.

Epidemiological transmission models have been developed for many animal hosts [7]–[11], and the relationship between metapopulation structure and the spread of directly transmitted diseases has been explored for group or herd-living animals [12], [13], as has the role of social structure in the spread of disease in primates and other animals. In particular, Thrall et al. [14] found that the mating system of marine mammals can alter the prevalence and extent of an outbreak of a sexually transmitted infection (STI) for different sexes in a population. These results are consistent with structured models for human STIs [15], [16]. Here we build on the large body of literature that has evolved for STIs in humans and couple host demography with social structure, to explore their combined role in the spread and initial epidemic persistence of infectious disease for nonhuman populations. Recent evidence suggests that key components of pathogen life history, such as the infectious and latent periods, are invariant across host taxa [17]. Because of this, we argue that within primate systems, disease dynamics will be mainly dictated by social interactions combined with demographic rates that are determined by body size [18].

### A Model of Social Interaction and Transmission

We modeled five major primate social systems: solitary, monogamous, unimale, multi-male, and fission-fusion (SO, MO, UM, MM and FF, respectively) [19], and created descriptive behavioral matrices, synthesized from the literature, of intergroup interactions for five disease transmission modes: sexual (STI), fecal-oral or local contamination (FO), aggressive interactions such as biting and scratching (AGGRO), direct aerosol (AERO), and vector transmitted disease (VEC) (Table 1). Even this simplified categorization of pathogen diversity and social organization leads to 25 different classes of interaction, of which only a few are redundant (Table S1).

**Table 1:** Glossary of Acronyms.

*Social Systems*	
SO	Solitary
MO	Monogamous
UM	Unimale
MM	Multi-male
FF	Fission-fusion
***Transmission modes***	
STI	Sexually transmitted infection
FO	Fecal-oral
AGGRO	Aggressive interactions
AERO	Aerosol, direct transmission
VEC	Vector transmission
***Model terms***	
SI	Susceptible-Infected
SIR	Susceptible-Infected-Recovered/Removed/Resistant
SIRS	Susceptible-Infected-Recovered/Removed/Resistant-Susceptible
WAIFW	Who Acquires Infection From Whom; matrix of interactions
***Demographic Stages***	
Inf	Infant class
sA_f_	Sub-adult female
sA_m_	Sub-adult male
A_f_	Adult female
A_m_	Adult male

Pathogen transmission depends on the rates at which hosts contact each other and these interactions occur at two nested spatial scales: within and between social group. These rates of interaction, and thus rates of pathogen transmission, will vary significantly between different social systems. Intergroup interactions may scale with group density within a landscape, but simple models for range overlap will mask age- (or stage-) specific behavioral interactions, such as territorial fights, so we included these in an explicit manner. Patterns of intergroup movement in primate social systems can be characterized into a few ‘canonical’ forms. For example, natal dispersal is a classic model of post-weaning dispersal of offspring, but it can differ between sexes such that males, females, or males and females, may disperse [20]–[23]. In fission-fusion societies, the intergroup exchange mechanism can be highly variable, in which single or multiple groups may leave one group and merge with another, or form new groups [20]. In this paper we focused on a male dispersal model, for ease of presentation, although our framework easily extends to many forms of intergroup interactions.

We use the SI (Susceptible-Infected) model for several reasons: if we look at high-profile African great ape disease outbreaks, particularly novel spillovers, Ebola is essentially SI, as is TB, and perhaps measles, although limited evidence for immunity has been seen in captivity. Some individuals may escape infection, but rarely is there recovery. This model framework could be expanded to an SIR model (Susceptible-Infected-Recovered), but SIR dynamics require large host populations for persistence – which may explain why we rarely see this in primates (other than humans). Furthermore, since groups have infrequent pathogen introductions, resistance and recovery (R in the model) would not build up sufficiently to create a buffering impact on the dynamics. An SIRS model (adding the return to Susceptible transition) would be essentially analogous to reducing body size and replacing the transition from R to S with a higher birth rate. Importantly, an SI model allows us to vary one unknown parameter (transmission) along its entire range to examine the dynamics of transmission and persistence. Future work will investigate the implications of other compartmental transmission models.

## Methods

### Characterizing Disease Establishment

The criterion for disease establishment, *R_0_*, describes the expected number of secondary cases created by an infected individual in a naïve population of hosts. The parameter is classically used as a threshold for disease establishment (*R_0_*>1), so we will use the relative magnitude of R_0_ as the basis to compare different types of pathogens in different social systems. In order to achieve this we use WAIFW (Who Acquires Infection from Whom) matrices to describe rates of interaction within and between different age and sex classes of the social system, whence different social systems will be defined here by WAIFW matrices with different relative rates of interaction, in ways that echo the different rates of interaction in different human societies [24], [25]. Here we develop hypothetical contact matrices for different social systems to obtain expressions for R_0_ after quantifying and explicitly including contacts within complex social networks, particularly for sexually transmitted diseases [15], [24], [26]–[34].

**Figure 1:**
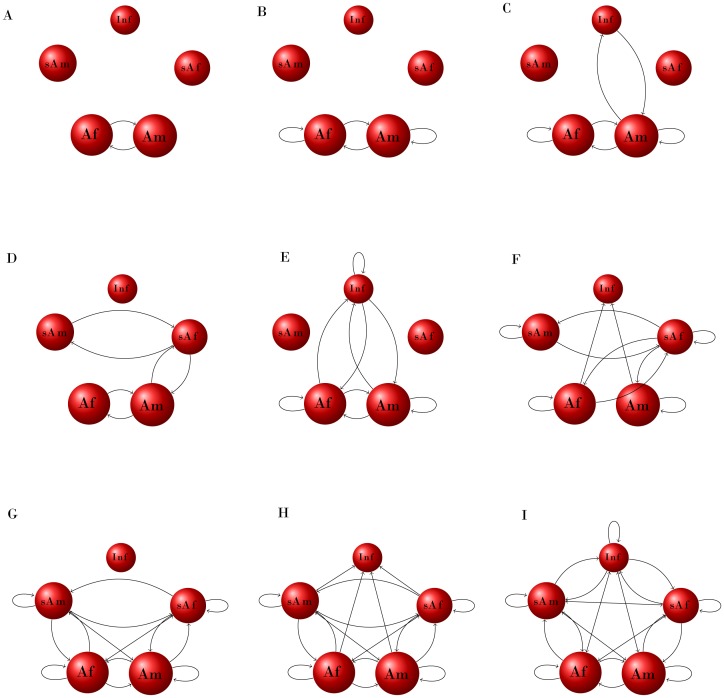
Nine unique networks representing within-group contact structure. These are network depictions of WAIFW (Who Acquired it From Whom) 5×5 matrices. The 5 age/sex classes (life-history stages) for which these are coded are infants (I), subadult females (sAf), subadult males (sAm), adult females (Af) and adult males (Am). The plots are as follows, with the transmission type, social system and number of edges, ***E***: **A**: STI – SO, MO; ***E*** = 2; **B**: AGGRO – SO; ***E*** = 4; **C**: AGGRO – MO; ***E*** = 6; **D**: STI – UM, MM; ***E*** = 6; **E**: FO– SO, MO; AERO – SO, MO; VEC – SO, MO; ***E*** = 9; **F**: AGGRO – UM, MM; ***E*** = 11; **G**: STI – FF; ***E*** = 16; H: AGGRO – FF; ***E*** = 20; **I**: The fully saturated contact matrix: FO – UM, MM, FF; AERO –UM, MM, FF; VEC – UM, MM, FF; ***E*** = 25.

Based on a compartmental demographic matrix model of five age-sex categories, we combined the social and behavioral matrices into WAIFW matrices, depicted in Figure 1, as nine unique digraphs (redundancy in routes of transmission reduces many of the 25 possible combinations to the 9 illustrated digraphs). We then integrated these into a compartmental demographic matrix model of five age-sex categories, with rules to emigrate, or associate and persist within the group, based on the five social system structures.

As our principle goal was to examine the role of social system (and body size) in constraining the establishment of virulent emerging pathogens in hosts with different social systems, we coupled our social system model with an SI disease transmission model. The models created could be expanded to include immunity, and initial explorations suggest that this can modify some of our conclusions with respect to persistence. In general, infections which lead to immunity require higher rates of transmission, which roughly scale with the duration of immunity. However, these can also create more complex epidemic dynamics such as those seen in classic studies of human diseases, including measles [35]–[38], and a later manuscript will explore some of these complexities for non-human primate populations. Here we focus on the constraints that social system and body size place on the initial establishment and impact of a non-immunizing infection.

We assume that infected individuals die at an enhanced rate of 5% per year. This mortality is in the low range of rates reported for outbreaks of flu-like respiratory infections in chimpanzees at Gombe National Park, Tanzania [1], [39], and is thus a proxy for a potential novel spillover disease from human populations. To demonstrate the differences induced by social interactions and demographic rates, over a range of transmissibility and intergroup movement rates, we modeled three life histories from a small, medium, and large primate, (S, M, L), using empirical life-history parameters (Table S2), providing us with three rates of demographic turnover. We review the implications of these model outputs and compare this with empirical information to explore model utility and application, particularly in a conservation context.

When we reduced the behavioral interactions and disease transmission modes to WAIFW matrices, as binary digraphs, we found that many of the combinations overlapped, generating nine unique graphs (Fig. 1). The initial combinations illustrate the myriad possible strengths of transmission between the age/gender life-history stages both within and between groups that could potentially occur in different social systems (Table S1). These nine unique binary digraphs (Fig. 1) obscure relative interaction strengths, but retain the within-group social system structure that is central to our model.

### Demographic Model

We created a ten-group matrix, comprising five-stage Lefkovitch matrices for within-group demography. The metapopulation is connected by movement of individuals between groups according to a specified probability *m* (described in a following section). Transitions between stages occur at rates specific to the length of the stage. Infants are assumed unsexed and make a transition to two sexes of subadults at a sex ratio of *σ* (which we typically assume to be 1), which, in turn, make transitions to adult males and females respectively. We assume female demographic dominance, meaning that the birthrate is proportional to the size of the adult female compartment. Births and transitions between stages are stochastic. Individual transitions within stages are assumed to follow independent Bernoulli trials, making the total number of transitions, per stage, per iteration, a binomial random number given by
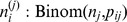
where 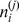 is the number of transitions from the *j*th to the *i*th stage, 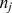 is the number of individuals in stage *j* and 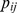 is the transition probability given by the Lefkovitch matrix.

Primates have a slower development rate than other mammals, postponing the age of sexual maturity and reproduction, and increasing the interbirth interval [18]. Larger primates have slower demographic turnover than smaller ones; we model this effect using a small primate with life-history characteristics based on Galago (*Galago spp.*) parameters (S), a mid-size primate, corresponding to a Leaf Monkey (*Presbytis spp.)* (M), and a large primate, based on Gorilla (*Gorilla gorilla*) parameters (L) (Table S2). Importantly, we wanted to obtain natural lifespan estimates for the life histories in question, as many primate life span estimates are derived from captivity, and likely to be much larger. To discuss the demographic turnover of the three exemplar primate life-histories, we use *T*, the generation time, formally described for a stationary population as:
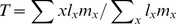
where *x* is the age class, *l_x_* is survivorship, and *m_x_* the age-specific fertility rate. Note that in the case of gorillas, the generation time will be longer than the average life span.

### Between Group Movement

We ran the model with subadult male dispersal connecting the metapopulation, as many primate social systems (at least among anthropoids) are based on female philopatry and male dispersal [22]. At each time step, the 100×1 next generation (NG) population vector (10 groups, 5 stages, 2 disease states) was updated after demographic and disease transitions were completeA uniform random draw on all groups determines which group will “receive” the migration. Again, we assume dispersal is governed by independent Bernoulli trials with movement probability *m* so that the number of migrants in the two disease states per time step is
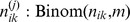
where 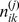 is the number of migrants to the *i*th class in disease state *k* (i.e., susceptible or infected) from the *j*th class.

### Contact Matrices

We used five primate social systems: solitary (SS), monogamous (MO), unimale (UM), multi-male (MM), and fission-fusion (FF) [19]. Demographic differences between the social systems were characterized by the parameters that determine interactions between the 5 life-stages in the demographic model above. We then added rates of within (intra-) group disease transmission for five types of transmission modes: sexual (STI), fecal-oral or local contamination (FO), aggressive interactions such as biting and scratching (AGGRO), direct aerosol (AERO), and vector transmitted disease (VEC) (Table 1).

The interactions were based on behavioral data, described for each age/sex class within the population, by social system and disease transmission type (Table S1). These comprise descriptions of intragroup and intergroup interactions, such as sexual encounters, mother-infant contact, migrations and dominance fights. These were described as digraphs of binary interactions between the five age/sex classes; some resultant matrices are identical, such as fecal-oral (FO), aerosol (AERO) and vector (VEC) transmission producing fully saturated intragroup contact matrices for UM, MM and FF social systems. This reduction to digraphs resulted in nine unique matrices (Fig. 1). We ordered these by size; the number of edges in the digraph, *E*, which is also a description of connectivity.

### Pathogen Transmission

The contact matrices and demographic models were coupled to a simple epidemiological compartmental SI (Susceptible-Infected) model [40]. Compartmental models track the dynamics of a disease as it moves through a population through time. *S* and *I* are the possible states of individuals in the population at a given time *t*, wherein they are either Susceptible or Infected and *S+I = N*.

The within-group update equations are given as follows:
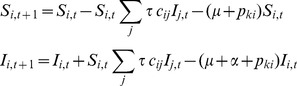
for *i,j,k* = 1,2,…5 where the index indicates the life-cycle stage, *τ* is the probability of transmission per contact, *C* with elements *c_ij_* is the WAIFW contact matrix, *μ* is the natural mortality rate, *α* is disease-induced mortality and *p* is the probability of transitioning to the next life-cycle stage.

We assumed the frequency dependent form of infection probability, to account for a within-group rate of infection [41], and additionally used the discrete time.
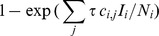
for the saturating probability of infection per time-step [12]. Calculations were implemented as a 100×100 transition matrix, with a 100×1 NG vector, such that within group infection and demography occurs prior to between-group dispersal, within each time-step.

Simulations were performed in Matlab 7 (Mathworks 1994–2006); code is provided in Text S1.

### Model Simulations

**Figure 2:**
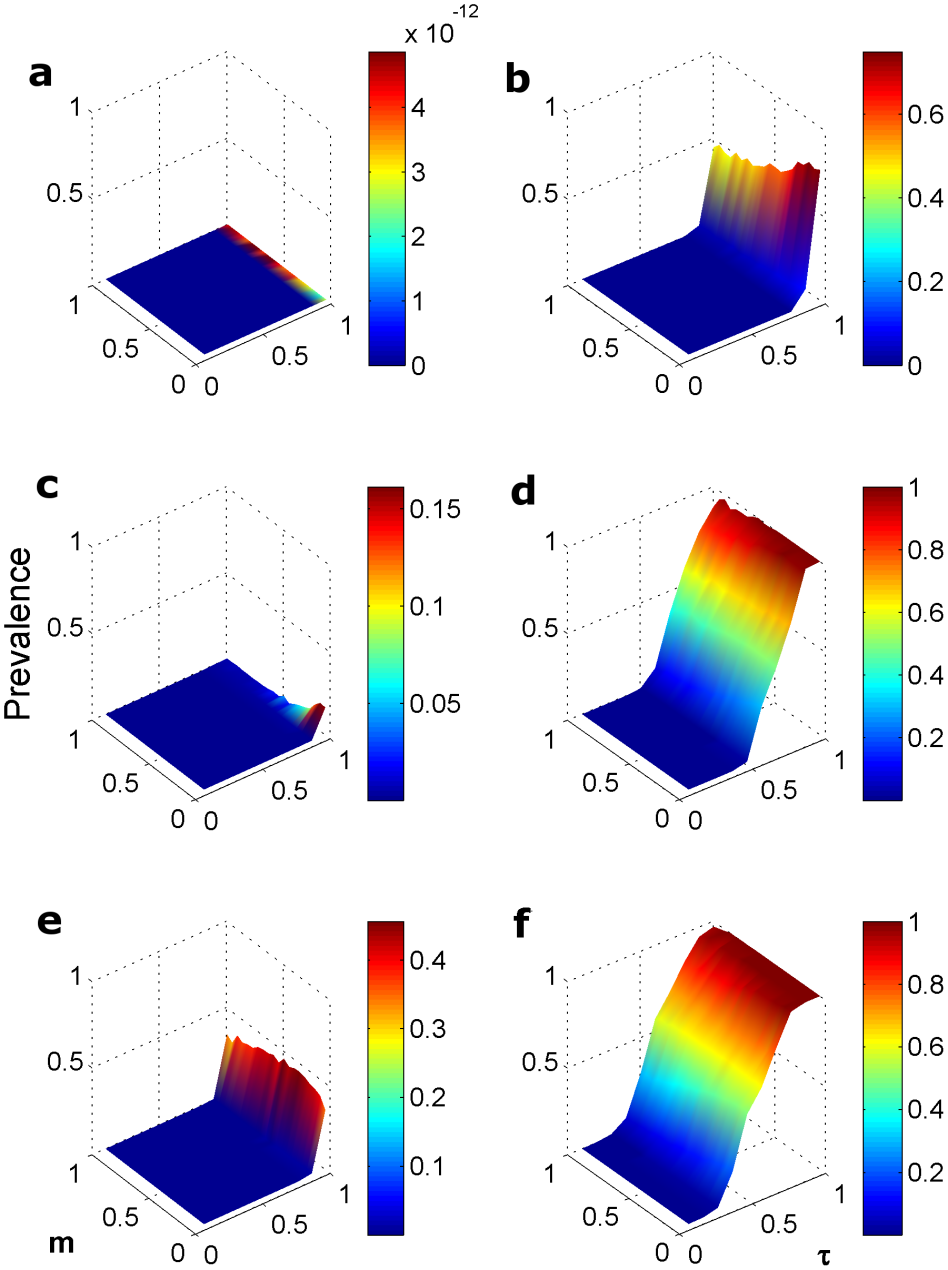
The mean prevalence (Z-axis, 0–1) of infection after 200 time steps over 100 iterations of the model for a unimale STI route and the fully saturated contact matrix. We vary the parameters movement, *m* (Y-axis, 0–1) and transmission, ***τ***. (X-axis, 0–1), and show the Small (a–b), Medium (c–d) and Large (e–f) demographic rates for the two contact structures depicting a unimale (UM) STI route, corresponding to matrix **D** (a, c, e) and the fully saturated contact matrix, **I**, (b, d, f), described in Figure 1. The full 27 results for matrices **A–I** demonstrating mean prevalence are given in **Figure S1a–c**.

To seed the model, we created an initial population vector of 10 groups, each with 5 individuals (one in each demographic class, all susceptible). To this we added an infectious sub-adult male in one group, simulating an immigration event or a single spillover into a group. We ran this model for three demographic scenarios, as given in Table S2, (S, M, L), and the nine unique contact structures (**A–I**) (Fig. 1). We varied the transmission probability *τ* over 0–1 and the movement probability, *m,* over 0–1 and ran the model for 100 iterations each of 200 time steps (years). The disease prevalence in the population over the range of *τ* and *m* are shown for each of the S, M, L demographic scenarios and for each unique contact matrix **A–I** (Fig. S1: a–c). Figure 2 shows the prevalence at 200 time steps for the S, M, L life histories and the simplest (**A**) and most connected (**I**) matrices.

**Figure 3:**
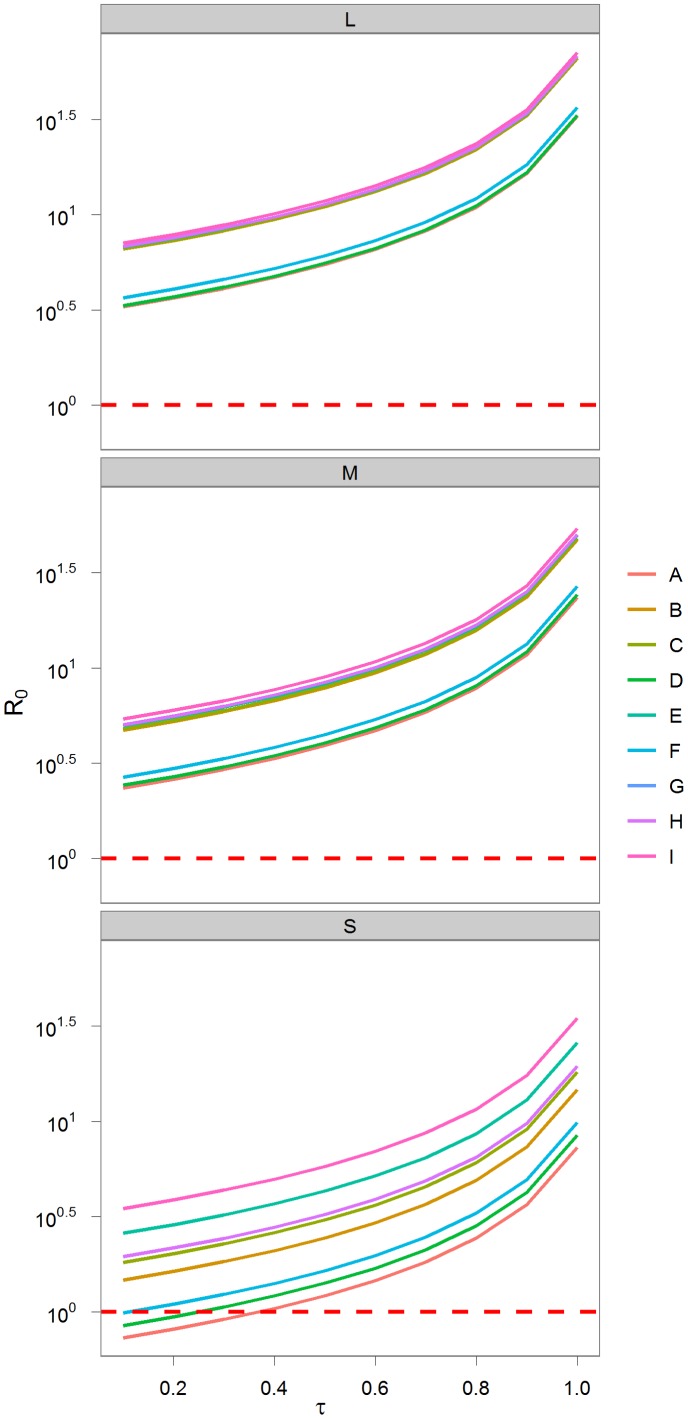
The range of values of *R_0_* for the large (L), medium (M), and small (S), primate demographic rates, across the range of transmission *τ* = (0–1). These are shown in order (**A–I**) of the increasing number of edges, *E*, in the contact structures. *R_0_* = 1 is shown with a hashed red line.

We used the next generation method [42]–[44], taking the dominant eigenvalue (spectral radius) of the product of the derived matrices *F* (the arising new susceptibles) and *V* ^−1^ (the inverse of the rates of loss from the infectious classes) to calculate *R_0_*, assuming the S, M, L life-histories and the 9 contact structures, over the range of *τ* (0–1 in 0.1 increments) used in the stochastic metapopulation simulation models (Fig. 3).

## Results

### Disease Establishment and Contact Structure

The rate at which new susceptibles are added to a population is central to pathogen establishment and persistence. Populations with the highest birth rates tend to have small body sizes and high mortality rates, and hence more rapid population turnover and shorter generation times than larger bodied species [18]. Our model revealed a non-intuitive trend: as population turnover decreased (increasing generation time), there was a general trend of increasing *R_0_*. This effect is most likely due to the frequency dependent transmission rates of the pathogens we are considering, as can be seen in Figure 3. *R_0_* did not increase directly with the number of edges in the within-group social network. For some social networks, underlying demographic structure impacts *R_0_* through stage duration and the number of individuals making it to the stage. For example, social structures E and F have 9 and 11 edges, respectively (Fig. 1); we might expect a corresponding increase in *R_0_*, but we see the opposite. The transmission modes described in E include FO, AERO and VEC transmission of parasites in SO and MO social systems, while F describes AGGRO interactions in UM and MM groups. Digraph E, with fewer edges, includes contacts for infants and adult females, whereas F is dominated by subadult and adult interactions. The demographic underpinnings of this suggest that in E, infants can become infected and remain infected, infecting others, while in F, they ‘escape’ infection, reducing overall epidemic spread. This emphasizes that social systems with frequent contacts with, and between, younger individuals will be more likely to maintain pathogens, than those with less contact between young and older animals. In this model, individuals are infected until they die, whether by natural or disease induced mortality. Initial exploration of a model including recovery shows that because the rate of recovery affects the degree to which infected individuals leave each sub-population or social group, and the duration of their infectiousness, the relationship between *R_0_* and population turnover can be significantly different.

### Disease Persistence and Stochasticity in a Socially-explicit Metapopulation

For large and medium sized primates, the classic well-mixed, fully saturated contact matrix (I, Fig. 1) enabled the pathogen to infect a large proportion of the population throughout much of the range of the movement and transmission parameter space (Fig. 2, D and F). This contrasts with the case for small primates where pathogen establishment is insensitive to movement rates when transmissibility is high (>0.60) (Fig. 2, B). Similarly, for small and medium sized primates, a unimale or multi-male social grouping sexual transmission mode (matrix D, Fig. 1) did not promote disease establishment and persistence except at very high transmissibility (Fig. 2, A, C). In the simulations for large bodied primates, the STI was able to persist at rates as high as 45% prevalence, given sufficiently high transmissibility (>0.90) and for most of the range of movement probabilities (>0.05) (Fig. 2, E). As for the *R_0_* estimates, fecal-oral, aerosol and vector transmission for solitary and monogamous groupings (matrix E) had bigger impacts on prevalence than aggressive interactions in unimale and multimale groups (matrix F) (Fig. S1).

The contrast in prevalence conferred by the demography of primates with different body sizes was most obvious for the STI and fully-saturated transmission (AERO and VEC). A counter-intuitive pattern of prevalence correlating with movement rate emerged for the small primates. While small primates have high population turnover (T; Table S2), for the fully saturated contact matrix, the disease reached higher prevalence when rates of intergroup movement were low. This suggests that there is a threshold of within-group infection before the disease can disperse to another group, presumably with an infected individual host (Fig. 2, lower panel).

The stochastic nature of infection and movement within a metapopulation occasionally gave results contrasting with our *R_0_* expectations for disease establishment. Only the smallest primate was predicted not to have pathogen persistence (*R_0_*<1), in a very few cases (Fig. 3). However, in our simulations there were many instances of low or zero prevalence over a range of transmission and movement values, for all three sizes modeled (Fig. S1). While a deterministic system will converge to *R_0_*, the accumulation of stochastic “extinctions” at each level of a metapopulation will lower disease persistence. It is therefore important in this type of modeling exercise to examine the role of stochastic events, such as movement and demographic events [45]. For example, if infection leads to mortality, higher *R_0_* can lead to faster ‘burn out’ of hosts, whereas lower *R_0_* (1.25>*R*_0_>1.0) can promote low-level, long term persistence [45], [46]. Transient dynamics of this type in a metapopulation are particularly relevant to management of pathogen outbreaks in populations of endangered primate species; our analysis suggests that slowing the spread of infection is more important than waiting for an endpoint, or natural die-off; as this might take a significant period of time.

## Discussion

Novel pathogen introduction has joined hunting and habitat loss as one of the leading threats to natural populations of primates, particularly the great apes [1]–[4]. The effect of anthropogenic pressure on remaining extant primate populations will create multifaceted disease risk potential. With decreasing and fragmented habitats, isolated small populations become far more vulnerable to local extinctions from disease [45], [47]–[50]. The phenomenon of spillover and spillback of zoonotic and human-based infectious diseases, particularly novel pathogens, into primate populations is likely to continue increasing with increasing population pressure [51]–[53]. As the rate of social system evolution in primates is probably much slower than the rate of pathogen adaptability to population structure, it is possible to predict characteristics of primates that predispose them to disease risk, using the framework we describe here. Although the epidemiological predictor *R_0_* encompasses risk in a simple threshold measure, our approach goes further and examines the transient dynamics and persistence of the pathogen. We incorporate social system and demography in a relatively straightforward way that provides important insights for a diversity of primate species.

The constraints on pathogens imposed by the interaction between social structure and host demography were most clear-cut for either the largest primates or the smallest. In small-bodied primates, high demographic turnover and fast maturation reduces the amount of time an individual is exposed to infected individuals within a group. This reduces the rate of infection of the rest of the population, as sub-adults can emigrate prior to infection. In contrast, larger-bodied primates spend more absolute time interacting within-group prior to emigration, increasing both their individual risk of infection and mortality, as well as that of their social group, and ultimately the overall population. Clearly a long-lived, large-bodied primate in a complex social system is likely to sustain an infectious disease with almost any transmission mode, provided sufficient intergroup contact exists in a social system. Their slow population turnover rates minimize the population resilience of great apes, and may explain why their populations have recently been significantly reduced by Ebola [2] and respiratory viruses [1], [4]. Everything we describe above suggests they are unlikely to recover to previous levels of abundance without intervention and prolonged periods of recovery time [1].

Habitat alteration may also be crucial in causing transient disruptions that allow pathogens to establish and diminish wild primate populations. We predict that fragmented populations are at increased extinction risk from disease due to smaller population size and a lack of rescue effects from the metapopulation [45]. However, there may be further impacts of fragmentation with less intuitive results. For example, in a study of red colobus (*Piliocolobus tephrosceles*), fragmentation of forest habitat caused resource scarcity, leading to fusion of foraging groups [54]. From the perspective of disease transmission, this fusing would increase contact rates, increasing disease vulnerability. If resource scarcity was contributing to nutritional stress and thus immunological stress, adding potential disease exposure would simply compound this vulnerability to disease invasion and persistence.

There is not yet sufficient data to quantify all the parameters necessary to make this a complete framework for infectious disease in all primates, although the availability of data is increasing (see Discussion S1). WAIFW matrices were useful in framing the contact structure, but without weighted interactions from empirical data, it is hard to quantify the true variation that would occur in the different age-gender classes. In our Discussion S1, we address some of the potential data sources and means to obtain appropriate parameters. There is a need for more field research into primate sociality and disease transmission, and increased attention to the results of physiological studies of captive primates. As wild populations of primates become increasingly threatened, and their environments impacted and changed by humans, it is important to have a framework in which to quantify the resulting contact structures and their effects. We suggest this study be used as a coarse level framework, assessing the potential disease risk to a population of known social system and demography. In essence, this is a metapopulation viability analysis model in which explicit disease impact can be assessed. As pointed out by Gerber et al. [55], disease should not simply be modeled as a single catastrophic event within a population. The demographically explicit nature of the structure of our model makes it flexible to the quality and quantity of information available for a primate species and a potential pathogen. As the timing of disease intervention in primates is likely to be during a reactive phase rather than proactive [1], understanding the transient dynamics will be crucial for disease control and population management.

While our approach relies on broad categories of social systems, we provide an initial framework for examining the interaction between social complexity and pathogen spread and persistence across primates that can be extended to more detailed primate social systems and other social vertebrates and to immunizing pathogens that will exhibit more complex patterns of persistence. We hope that this basic framework spurs further studies, particularly incorporating spatially explicit information into models of primate disease potential. In this way, conservation of surviving primate populations and assessment of potential Emerging Infectious Diseases (EIDs) can be highlighted and preemptive disease control strategies developed for future outbreaks.

## Supporting Information

Figure S1 **The mean prevalence (Z-axis, 0–1) of infection after 200 time steps over 100 iterations of the model, varying the parameters** ***m*** **(Y-axis, 0–1) and** ***τ*** **(X-axis, 0–1).** This is shown for the a. Small, b. Medium and c. Large demographic rates (Table S2). Within each of these, the 9 unique contact structures from Figure 1 are demonstrated, labeled A–I in order of the number of ordered pairs (or size of the graph, E – A:2, B:4, C:5, D:6, E:9, F:11, G:16, H:20, I:25)
(TIF)

Table S1 **Contact structure for intragroup (matrices1–5) and intergroup (matrices 6–10) interactions given social system (rows) and transmission mode (columns).** The 5 age/sex classes (life-history stages) for which these are coded are infants (I), subadult females (sA_f_), subadult males (sA_m_), adult females (A_f_) and adult males (A_m_). Vertical transmission is written (A_f_i). The infectious parameter β is used for intimate contact transmission and β_v_ for vector transmission. β_FIGHT_ implies the disease risk from an aggressive interaction, and β_GROOM_ is the risk during a grooming interaction. In matrices 6–10 where intergroup interactions are described, dichotomous interactions for emigrating (ε and immigrating (i) individuals can occur, and the direction of these is described using a 1, 2 from-to group notation. Δ is used for vector transmission between groups as a variable for distance between the two groups.
(DOCX)

Table S2 **Life-history parameters for Small, Medium and Large (S, M, L) primates.**
(DOC)

Text S1 **Matlab model code.**
(TXT)

Discussion S1 **From theory to reality: how can we establish and understand contact rates for these transmission modes?**
(DOCX)

## References

[1] Ryan SJ, Walsh PD (2011) Consequences of Non-Intervention for Infectious Disease in African Great Apes. PLoS ONE 6: e29030. 22216162 10.1371/journal.pone.0029030 PMC3245243

[2] Walsh PD, Abernethy KA, Bermejo M, Beyersk R, De Wachter P, et al (2003) Catastrophic ape decline in western equatorial Africa. Nature 422: 611–614. 12679788 10.1038/nature01566

[3] Bermejo M, Rodriguez-Teijeiro JD, Illera G, Barroso A, Vila C, et al.. (2006) Ebola Outbreak Killed 5000 Gorillas. Science 314: 1564–. 10.1126/science.1133105 17158318

[4] Kondgen S, Kuhl H, N’Goran PK, Walsh PD, Schenk S, et al (2008) Pandemic human viruses cause decline of endangered great apes. Current Biology 18: 260–264. 18222690 10.1016/j.cub.2008.01.012

[5] Harvey PH, Martin RD, Clutton-Brock TH (1987) Life histories in comparative perspective. In: Smutts BB, Cheney DL, Seyfarth RM, Wrangham RW, Struhsaker TT, editors. Primate Societies. Chicago: Chicago University Press. 181–196.

[6] Roberts M, Heesterbeek H (1993) Bluff your way in epidemic models. Trends in Microbiology 1: 343–348. 8162424 10.1016/0966-842x(93)90075-3

[7] Dobson AP, Hudson PJ (1992) Regulation and stability of a free-living host - parasite system: Trichostrongylus tenuis in red grouse. II. Population models. Journal of Animal Ecology 61: 487–498.

[8] Grenfell BT, Dobson AP (1995) Ecology of Infectious Diseases in Natural Populations. New York: Cambridge University Press.

[9] Heesterbeek JA, Roberts MG (1995) Mathematical models for microparasites of wildlife. In: Grenfell BT, Dobson AP, editors. Ecology of Infectious Diseases in Natural Populations. Cambridge: Cambridge University Press. 90–122.

[10] Dobson A, Foufopoulos J (2001) Emerging infectious pathogens of wildlife. Philosophical Transactions of the Royal Society of London Series B-Biological Sciences 356: 1001–1012. 10.1098/rstb.2001.0900 PMC1088495 11516378

[11] Holt RD, Dobson AP, Begon M, Bowers RG, Schauber EM (2003) Parasite establishment in host communities. Ecology Letters 6: 837–842.

[12] Cross PC, Johnson PLF, Lloyd-Smith JO, Getz WM (2007) Utility of Ro as a predictor of disease invasion in structured populations. Journal of the Royal Society Interface 4 315: 324. 10.1098/rsif.2006.0185 PMC2359845 17251146

[13] Cross PC, Lloyd-Smith JO, Johnson PLF, Getz WM (2005) Duelling timescales of host movement and disease recovery determine invasion of disease in structured populations. Ecology Letters 8: 587–595.

[14] Thrall PH, Antonovics J, Dobson AP (2000) Sexually transmitted diseases in polygynous mating systems: prevalence and impact on reproductive success. Proceedings of the Royal Society Biological Sciences Series B 267: 1555–1563. 10.1098/rspb.2000.1178 PMC1690713 11007332

[15] Morris M (1993) Epidemiology and social networks: modeling structured diffusion. Sociological Methods and Research 22: 99–126.

[16] Jones JH (2008) Sexually transmitted infections as biomarkers of cultural behavior. In: Brown M, editor. Explaining Culture Scientifically. Seattle: University of Washington Press.

[17] Cable JM (2007) The Allometry of Host-Pathogen Interactions. PLoS ONE 2. 10.1371/journal.pone.0001130 PMC2042517 17987117

[18] Charnov EL, Berigan D (1993) Why do female primates have such long lifespans and so few babies? or Life in the slow lane. Evolutionary Anthropology: 191–194.

[19] Richard AF (1985) Primates in nature. New York: W.H. Freeman and Company. 588 p.

[20] Alberts SC, Altmann J (1995) Balancing costs and opportunities - dispersal in male baboons. American Naturalist 145: 279–306.

[21] Wrangham R (1979) On the evolution of ape social systems. Social Science Information 18: 334–368.

[22] Wrangham RW (1980) An ecological model of female-bonded primate groups. Behaviour 75: 262–300.

[23] Pusey AE (1992) The primate perspective on dispersal. In: Stenseth NC, Lidicker WZ, editors. Animal dispersal: small mammals as a model. New York, NY: Chapman and Hall. 243–259.

[24] Edmunds WJ, Ocallaghan CJ, Nokes DJ (1997) Who mixes with whom? A method to determine the contact patterns of adults that may lead to the spread of airborne infections. Proceedings of the Royal Society Biological Sciences Series B 264: 949–957. 10.1098/rspb.1997.0131 PMC1688546 9263464

[25] Mossong J, Hens N, Jit M, Beutels P, Auranen K, et al (2008) Social Contacts and Mixing Patterns Relevant to the Spread of Infectious Diseases. PLoS Med 5: e74. 18366252 10.1371/journal.pmed.0050074 PMC2270306

[26] Corner LAL, Pfeiffer DU, Morris RS (2003) Social-network analysis of Mycobacterium bovis transmission among captive brushtail possums (*Trichosurus vulpecula*). Preventive Veterinary Medicine 59: 147–167. 12809760 10.1016/s0167-5877(03)00075-8

[27] Morris M (1991) A log-linear modeling framework for selective mixing. Mathematical Biosciences 107: 349–377. 1806123 10.1016/0025-5564(91)90014-a

[28] Morris M (1995) Data driven network models for the spread of disease. In: Mollison D, editor. Epidemic Models: their structure and relation to data. Cambridge: Cambridge University Press.

[29] Morris M (1996) Behaviour change and non-homogenous mixing. In: Isham V, Medley GF, editors. Models for Infectious Human Diseases. Cambridge: Cambridge University Press.

[30] Boots M, Sasaki A (1999) ‘Small worlds’ and the evolution of virulence: infection occurs locally and at a distance. Proceedings of the Royal Society Biological Sciences Series B 266: 1933–1938. 10.1098/rspb.1999.0869 PMC1690306 10584335

[31] Ferguson NM, Garnett GP (2000) More realistic models of sexually transmitted disease transmission dynamics - Sexual partnership networks, pair models, and moment closure. Sexually Transmitted Diseases 27: 600–609. 11099075 10.1097/00007435-200011000-00008

[32] Eames KTD, Keeling MJ (2002) Modeling dynamic and network heterogeneities in the spread of sexually transmitted diseases. Proceedings of the National Academy of Sciences of the United States of America 99: 13330–13335. 12271127 10.1073/pnas.202244299 PMC130633

[33] Eames KTD, Keeling MJ (2003) Contact tracing and disease control. Proceedings of the Royal Society Biological Sciences Series B 270: 2565–2571. 10.1098/rspb.2003.2554 PMC1691540 14728778

[34] Eames KTD (2008) Modelling disease spread through random and regular contacts in clustered populations. Theoretical Population Biology 73: 111. 10.1016/j.tpb.2007.09.007 18006032

[35] Grenfell B (1992) Chance and chaos in measles dynamics. Journal of the Royal Statistical Society Series B (Methodological): 383–398.

[36] Bartlett MS (1957) Measles Periodicity and Community Size. Journal of the Royal Statistical Society Series A (General) 120: 48–70.

[37] Anderson RM, May RM (1979) Population biology of infectious diseases: part I. Nature. 280: 361–366. 10.1038/280361a0 460412

[38] Cliff AD, Ord JK (1978) Forecasting the progress of an epidemic. In: Martin RL, Thrift NJ, Bennett RJ, editors. Towards the Dynamic Analysis of Spatial Systems. London: Pion. 191–204.

[39] Williams JM, Lonsdorf EV, Wilson ML, Schumacher-Stankey J, Goodall J, et al (2008) Causes of death in the Kasekela chimpanzees of Gombe National Park, Tanzania. American Journal of Primatology 70: 766–777. 18506732 10.1002/ajp.20573

[40] Anderson RM, May RM (1991) Infectious diseases of humans: dynamics and control. Oxford: Oxford University Press. 757 p.

[41] Getz WM, Pickering J (1983) Epidemic models: thresholds and population regulation. American Naturalist 121: 892–898.

[42] Diekmann O, Heesterbeek JAP, Metz JAJ (1990) On the Definition and the Computation of the Basic Reproduction Ratio R0 in Models for Infectious-Diseases in Heterogeneous Populations. Journal of Mathematical Biology 28: 365–382. 2117040 10.1007/BF00178324

[43] van den Driessche P, Watmough J (2002) Reproduction numbers and sub-threshold endemic equilibria for compartmental models of disease transmission. Mathematical Biosciences 180: 29–48. 12387915 10.1016/s0025-5564(02)00108-6

[44] Hefferman JM, Smith RJ, LM W (2005) Perspectives on the basic reproductive ratio. Journal of the Royal Society Interface 2: 281–293. 10.1098/rsif.2005.0042 PMC1578275 16849186

[45] McCallum H, Dobson A (2006) Disease and connectivity. In: Crooks KR, Sanjayan M, editors. Connectivity Conservation. Cambridge: Cambridge Books Online. Cambridge University Press.

[46] Craft ME, Hawthorne PL, Packer C, Dobson AP (2008) Dynamics of a multihost pathogen in a carnivore community. Journal of Animal Ecology 77: 1257–1264. 18540966 10.1111/j.1365-2656.2008.01410.x

[47] Hess GR (1996) Linking extinction to connectivity and habitat destruction in metapopulation models. American Naturalist 148: 226–236.

[48] Hess G (1996) Disease in metapopulation models: Implications for conservation. Ecology 77: 1617–1632.

[49] Hess GR (1994) Conservation Corridors and Contagious Disease: A Cautionary Note. Conservation Biology 8: 256–262.

[50] McCallum H, Dobson A (2002) Disease, habitat fragmentation and conservation. Proceedings of the Royal Society Biological Sciences Series B 269: 2041–2049. 10.1098/rspb.2002.2079 PMC1691124 12396504

[51] Daszak P, Cunningham AA, Hyatt AD (2000) Wildlife ecology - Emerging infectious diseases of wildlife - Threats to biodiversity and human health. Science 287: 443–449. 10642539 10.1126/science.287.5452.443

[52] Wolfe ND, Daszak P, Kilpatrick AM, Burke DS (2005) Bushmeat hunting, deforestation, and prediction of zoonoses emergence. Emerging Infectious Diseases 11. 10.3201/eid1112.040789 PMC3367616 16485465

[53] Jones KE, Patel NG, Levy MA, Storeygard A, Balk D, et al (2008) Global trends in emerging infectious diseases. Nature 451: 990–993. 18288193 10.1038/nature06536 PMC5960580

[54] Marshall AR, Topp-Jorgensen JE, Brink H, Fanning E (2005) Monkey abundance and social structure in two high-elevation forest reserves in the Udzungwa Mountains of Tanzania. International Journal of Primatology 26: 127–145.

[55] Gerber LR, McCallum H, Lafferty KD, Sabo JL, Dobson A (2005) Exposing extinction risk analysis to pathogens: is disease just another form of density dependence? Ecological Applications 15: 1402–1414.

