# Supplementary figures and images for: Interactions between Social Structure, Demography, and Transmission Determine Disease Persistence in Primates
Source: PLoS One. 2013 Oct 18;8(10):e76863. doi: 10.1371/journal.pone.0076863 (PMC3800049; doi:10.1371/journal.pone.0076863)

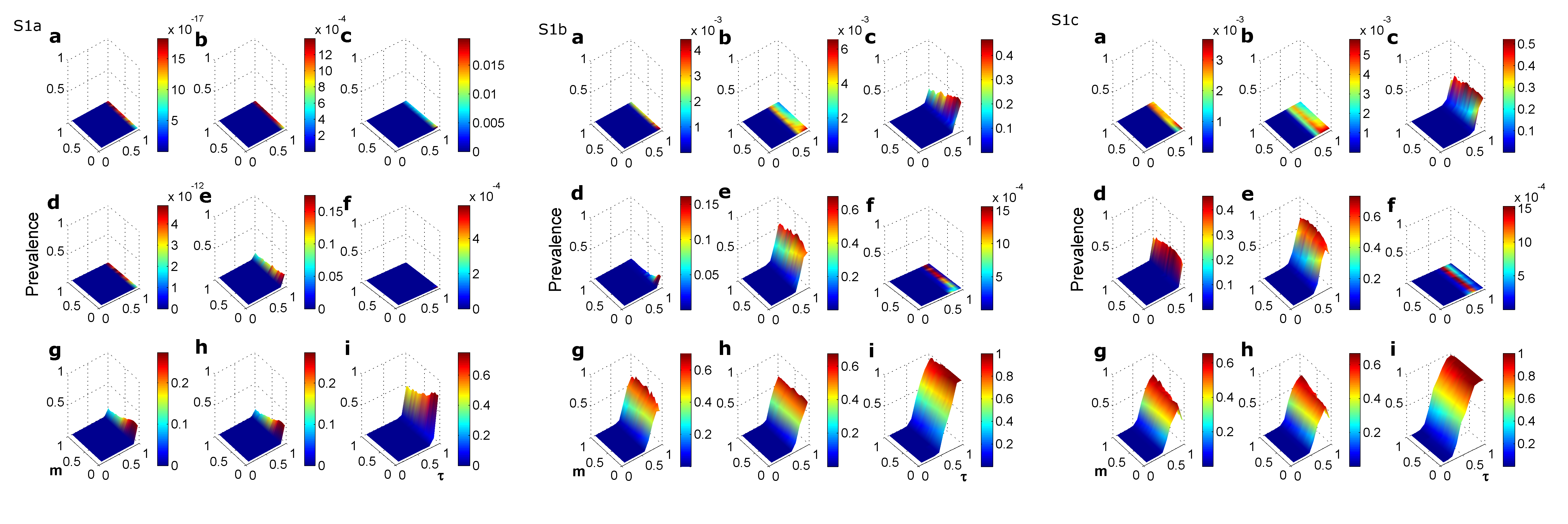

Supplement: Figure S1 — The mean prevalence (Z-axis, 0–1) of infection after 200 time steps over 100 iterations of the model, varying the parameters m (Y-axis, 0–1) and τ (X-axis, 0–1). This is shown for the a. Small, b. Medium and c. Large demographic rates (Table S2). Within each of these, the 9 unique contact structures from Figure 1 are demonstrated, labeled A–I in order of the number of ordered pairs (or size of the graph, E – A:2, B:4, C:5, D:6, E:9, F:11, G:16, H:20, I:25) (TIF) [file pone.0076863.s001.tif]
