# Supplementary material for: Interactions between Social Structure, Demography, and Transmission Determine Disease Persistence in Primates
Source: PLoS One. 2013 Oct 18;8(10):e76863. doi: 10.1371/journal.pone.0076863 (PMC3800049; doi:10.1371/journal.pone.0076863)
Supplement: Table S1 — Contact structure for intragroup (matrices1–5) and intergroup (matrices 6–10) interactions given social system (rows) and transmission mode (columns). The 5 age/sex classes (life-history stages) for which these are coded are infants (I), subadult females (sAf), subadult males (sAm), adult females (Af) and adult males (Am). Vertical transmission is written (Afi). The infectious parameter β is used for intimate contact transmission and βv for vector transmission. βFIGHT implies the disease risk from an aggressive interaction, and βGROOM is the risk during a grooming interaction. In matrices 6–10 where intergroup interactions are described, dichotomous interactions for emigrating (ε and immigrating (i) individuals can occur, and the direction of these is described using a 1, 2 from-to group notation. Δ is used for vector transmission between groups as a variable for distance between the two groups. (DOCX) [file pone.0076863.s002.docx]

**Table S1**

**1. Infant Intragroup Transmission Possibilities**

|  | STI | Fecal Oral | Aggression/Grooming | Aerosol | Vector |
| --- | --- | --- | --- | --- | --- |
| FF  *(Bonobos)* | *Bonobo sex:*  I[(βA_f_)+ (βA_m_) +(βsA_m_) + (βsA_m_)+ (βI)]  *Vertical:* I(βA_f_i) | I[(βA_f_)+ (βA_m_) +(βsA_m_) + (βsA_m_) + (βI)] | 0 | I[(βA_f_)+ (βA_m_) +(βsA_m_) + (βsA_m_) + (βI)] | I[(β_v_A_m_) + (β_v_A_f_) + (β_v_sA_m_) + (β_v_sA_f_)+ (β_v_I)] |
| MM | *Vertical:* I(βA_f_i) | I[(βA_f_)+ (βA_m_) +(βsA_m_) + (βsA_m_) + (βI)] | 0 | I[(βA_f_)+ (βA_m_) +(βsA_m_) + (βsA_m_) + (βI)] | I[(β_v_A_m_) + (β_v_A_f_) + (β_v_sA_m_) + (β_v_sA_f_)+ (β_v_I)] |
| UM | *Vertical:* I(βA_f_i) | I[(βA_f_)+ (βA_m_) +(βsA_m_) + (βsA_m_) + (βI)] | 0 | I[(βA_f_)+ (βA_m_) +(βsA_m_) + (βsA_m_) + (βI)] | I[(β_v_A_m_) + (β_v_A_f_) + (β_v_sA_m_) + (β_v_sA_f_)+ (β_v_I)] |
| MO | *Vertical:* I(βA_f_i) | I[(βA_f_)+ (βA_m_) + (βI)] | 0 | I[(βA_f_)+ (βA_m_) + (βI)] | I[(β_v_A_m_) + (β_v_A_f_) + (β_v_I)] |
| SO  *(infants still in the natal group)* | Vertical: I(βAfi) | I[(βA_f_)+ (βA_m_) + (βI)] | 0 | I[(βA_f_)+ (βA_m_) + (βI)] | I[(β_v_A_m_) + (β_v_A_f_) + (β_v_I)] |

**2. Subadult Female Intragroup Transmission Possibilities**

|  | STI | Fecal Oral | Aggression/Grooming | Aerosol | Vector |
| --- | --- | --- | --- | --- | --- |
| FF  *(Bonobos)* | sA_f_[(βA_f_)+ (βA_m_) +(βsA_m_) + (βsA_m_)+ (βI)] | sA_f_[(βA_f_)+ (βA_m_) +(βsA_m_) + (βsA_m_)+ (βI)] | *Dominance fights*:  sA_f_ [β_FIGHT_ (A_m_ + A_f_ + sA_m_ + sA_f_)]  -------------------------------------------  *Allomothering and grooming favors:*  sA_f_ [β_GROOM_ (A_m_ + A_f_ + sA_f_)] | sA_f_[(βA_f_)+ (βA_m_) +(βsA_m_) + (βsA_m_)+ (βI)] | I[(β_v_A_m_) + (β_v_A_f_) + (β_v_sA_m_) + (β_v_sA_f_)+ (β_v_I)] |
| MM | sA_f_[(βA_m_) *+(βsA_m_)*]  *Subadult males may copulate occasionally* | sA_f_[(βA_f_)+ (βA_m_) +(βsA_m_) + (βsA_m_)+ (βI)] | *Dominance fights:*  sA_f_ [β_FIGHT_ (A_m_ + A_f_ + sA_m_ + sA_f_)]  -------------------------------------------  *Allomothering and grooming favors:*  sA_f_ [β_GROOM_ (A_m_ + A_f_ + sA_f_)] | sA_f_[(βA_f_)+ (βA_m_) +(βsA_m_) + (βsA_m_)+ (βI)] | I[(β_v_A_m_) + (β_v_A_f_) + (β_v_sA_m_) + (β_v_sA_f_)+ (β_v_I)] |
| UM | sA_f_[(βA_m_) *+(βsA_m_)*]  *Subadult males may copulate, but tend to migrate instead* | sA_f_[(βA_f_)+ (βA_m_) +(βsA_m_) + (βsA_m_)+ (βI)] | *Dominance fights:*  sA_f_ [β_FIGHT_ (A_m_ + A_f_ + sA_m_ + sA_f_)]  -------------------------------------------  *Allomothering and grooming favors:*  sA_f_ [β_GROOM_ (A_m_ + A_f_ + sA_f_)] | sA_f_[(βA_f_)+ (βA_m_) +(βsA_m_) + (βsA_m_)+ (βI)] | I[(β_v_A_m_) + (β_v_A_f_) + (β_v_sA_m_) + (β_v_sA_f_)+ (β_v_I)] |
| MO | 0 | 0 | 0 | 0 | 0 |
| SO | 0 | 0 | 0 | 0 | 0 |

#### 3. Subadult Male Intragroup Transmission Possibilities

|  | STI | Fecal Oral | Aggression/Grooming | Aerosol | Vector |
| --- | --- | --- | --- | --- | --- |
| FF  *(Bonobos)* | sA_m_[(βA_f_)+ (βA_m_) +(βsA_m_) + (βsA_m_)+ (βI)] | sA_m_[(βA_f_)+ (βA_m_) +(βsA_m_) + (βsA_m_)+ (βI)] | *Dominance fights:*  A_m_ [β_FIGHT_ (A_m_ + A_f_ + sA_m_ + sA_f_ + I)  -------------------------------------------  *Grooming favors:*  A_m_ [β_GROOM_ (A_m_ + A_f_ + sA_m_ + sA_f_)] | sA_m_[(βA_f_)+ (βA_m_) +(βsA_m_) + (βsA_m_)+ (βI)] | sA_m_[(β_v_A_m_) + (β_v_A_f_) + (β_v_sA_m_) + (β_v_sA_f_)+ (β_v_I)] |
| MM | 0 | sA_m_[(βA_f_)+ (βA_m_) +(βsA_m_) + (βsA_m_)+ (βI)] | *Dominance fights:*  A_m_ [β_FIGHT_ (A_m_ + sA_m_)]  -------------------------------------------  *Grooming favors:*  A_m_ [β_GROOM_ (A_m_ + A_f_ + sA_m_ + sA_f_)] | sA_m_[(βA_f_)+ (βA_m_) +(βsA_m_) + (βsA_m_)+ (βI)] | sA_m_[(β_v_A_m_) + (β_v_A_f_) + (β_v_sA_m_) + (β_v_sA_f_)+ (β_v_I)] |
| UM | 0 | sA_m_[(βA_f_)+ (βA_m_) +(βsA_m_) + (βsA_m_)+ (βI)] | *Dominance fights:*  A_m_ [β_FIGHT_ (A_m_ + sA_m_)]  -------------------------------------------  *Grooming favors:*  A_m_ [β_GROOM_ (A_m_ + A_f_ + sA_m_ + sA_f_)] | sA_m_[(βA_f_)+ (βA_m_) +(βsA_m_) + (βsA_m_)+ (βI)] | sA_m_[(β_v_A_m_) + (β_v_A_f_) + (β_v_sA_m_) + (β_v_sA_f_)+ (β_v_I)] |
| MO | 0 | 0 | 0 | 0 | 0 |
| SO | 0 | 0 | 0 | 0 | 0 |

#### 4. Adult Female Intragroup Transmission Possibilities

|  | | STI | Fecal Oral | Aggression/Grooming | Aerosol | Vector |
| --- | --- | --- | --- | --- | --- | --- |
| FF  *(Bonobos)* | | A_f_[(βA_f_)+ (βA_m_) +(βsA_m_) + (βsA_m_)+ (βI)] | A_f_[(βA_f_)+ (βA_m_) +(βsA_m_) + (βsA_m_)+ (βI)] | *Dominance fights and infant protection:*  A_f_ [β_FIGHT_ (A_m_ + A_f_ + sA_m_ + sA_f_)]  -------------------------------------------  *Allomothering and grooming favors:*  A_f_ [β_GROOM_ (A_m_ + A_f_ + sA_m_ + sA_f_)] | A_f_[(βA_f_)+ (βA_m_) +(βsA_m_) + (βsA_m_)+ (βI)] | A_f_[(β_v_A_m_) + (β_v_A_f_) + (β_v_sA_m_) + (β_v_sA_f_)+ (β_v_I)] |
| MM | | *Occasional (rare) subadult copulation:*  A_f_[(βA_m_) *+(βsA_m_)*] | A_f_[(βA_f_)+ (βA_m_) +(βsA_m_) + (βsA_m_)+ (βI)] | *Dominance fights and infant protection:*  A_f_ [β_FIGHT_ (A_m_ + A_f_ + sA_m_ + sA_f_)]  -------------------------------------------  *Allomothering and grooming favors:*  A_f_ [β_GROOM_ (A_m_ + A_f_ + sA_f_)] | A_f_[(βA_f_)+ (βA_m_) +(βsA_m_) + (βsA_m_)+ (βI)] | A_f_[(β_v_A_m_) + (β_v_A_f_) + (β_v_sA_m_) + (β_v_sA_f_)+ (β_v_I)] |
| UM | | *Occasional (rare) subadult copulation:*  A_f_[(βA_m_) *+(βsA_m_)*] | A_f_[(βA_f_)+ (βA_m_) +(βsA_m_) + (βsA_m_)+ (βI)] | *Dominance fights and infant protection:*  A_f_ [β_FIGHT_ (A_m_ + A_f_ + sA_m_ + sA_f_)]  -------------------------------------------  *Allomothering and grooming favors:*  A_f_ [β_GROOM_ (A_m_ + A_f_ + sA_f_)] | A_f_[(βA_f_)+ (βA_m_) +(βsA_m_) + (βsA_m_)+ (βI)] | A_f_[(β_v_A_m_) + (β_v_A_f_) + (β_v_sA_m_) + (β_v_sA_f_)+ (β_v_I)] |
| MO | A_f_[(βA_m_)] | A_f_[(βA_f_)+ (βA_m_) + (βI)] | Few or no agonistic encounters  -------------------------------------------  *Grooming:*  A_f_ [β_GROOM_ (A_m_ )] | A_f_[(βA_f_)+ (βA_m_) + (βI)] | A_f_[(β_v_A_m_) + (β_v_A_f_) + (β_v_I)] |  |
| SO  *Encounters only during breeding seasons* | | A_f_[(βA_m_)] | A_f_[(βA_m_)+ (βI)] | Few or no agonistic encounters  -------------------------------------------  Grooming:  A_f_ [β_GROOM_(A_m_ )] | A_f_[(βA_f_)+ (βA_m_) + (βI)] | A_f_[(β_v_A_m_) + (β_v_A_f_) + (β_v_I)] |

**5. Adult Male Intragroup Tranmission Possibilities**

|  | STI | Fecal Oral | Aggression/Grooming | Aerosol | Vector |
| --- | --- | --- | --- | --- | --- |
| FF  *(Bonobos)* | A_m_[(βA_f_)+ (βA_m_) +(βsA_m_) + (βsA_m_)+ (βI)] | A_m_[(βA_m_) +(βA_f_) + (βsA_m_) + (βsA_f_) + (βI)] | A_m_ [β_FIGHT_ (A_m_ + A_f_ + sA_m_ + sA_f_ + I)]  -------------------------------------------  *Grooming favors:*  A_m_ [β_GROOM_ (A_m_ + A_f_ + sA_m_ + sA_f_ + I)] | A_m_[(βA_m_) + (βA_f_) + (βsA_m_) + (βsA_f_)+ (βI)] | A_m_[(β_v_A_m_) + (β_v_A_f_) + (β_v_sA_m_) + (β_v_sA_f_)+ (β_v_I)] |
| MM | A_m_[(βA_f_)+ +(βsA_f_)] | A_m_[(βA_m_) + (βAf) + (βsA_m_) +(βsA_f_)+ (βI)] | A_m_ [β_FIGHT_ (A_m_ + A_f_ + sA_m_ + sA_f_ + I)]  -------------------------------------------  *Grooming favors:*  A_m_ [β_GROOM_ (A_m_ + A_f_ + sA_m_ + sA_f_ + I)] | A_m_[(βA_m_) + (βA_f_) + (βsA_m_) + (βsA_f_)+ (βI)] | A_m_[(βA_m_) + (βA_f_) + (βsA_m_) + (βsA_f_)+ (βI)] |
| UM | A­_m_ [(βA_f_) +(βsA_f_)] | A_m_[(βA_m_) + (βA_f_) + (βsA_m_) + (βsA_f_)+ (βI)] | A_m_ [β_FIGHT_ (A_m_ + A_f_ + sA_m_ + sA_f_ + I)]  -------------------------------------------  *Grooming favors:*  A_m_ [β_GROOM_ (A_f_ + sA_m_ + sA_f_ + I)] | A_m_[(βA_m_) + (βA_f_) + (βsA_m_) + (βsA_f_)+ (βI)] | A_m_[(βA_m_) + (βA_f_) + (βsA_m_) + (βsA_f_)+ (βI)] |
| MO | A_m_[(βA_f_)] | A_m_[(βAf) +(βI)] | 0  -------------------------------------------  *Grooming favors:*  A_m_ [β_GROOM_ (A_f_ + sA_m_ + sA_f_ + I)] | A_m_[(βA_m_) + (βA_f_) + (βI)] | A_m_[(βA_m_) + (βA_f_) + (βI)] |
| SO  *Encounters only during breeding seasons* | A_m_[(βA_f_)] | A_m_[(βA_f_)+ (βI)] | 0 | 0 | A_m_[(β_v_A_f_)] |

**6. Infant Intergroup Transmission Possibilities**

|  | STI | Fecal Oral | Aggression/Grooming | Aerosol | Vector |
| --- | --- | --- | --- | --- | --- |
| FF  *(Bonobos)* | 0 | I[(βA_m_)_1,2_ + (βA_f_)_1,2_ + (βsA_m_)_1,2_ + (βsA_f_)_1,2_ + (βI) _1,2_] | *Infanticidal takeovers:*  I_1_[(βA_m_) + (βA_f_) + (βsA_m_)] _2_ | 0 | I_1_Δ[(β_v_A_m_) + (β_v_A_f_) + (β_v_sA_m_) + (β_v_sA_f_)+ (β_v_I)]_2_ |
| MM | 0 | I[(βA_m_)_1,2_ + (βA_f_)_1,2_ + (βsA_m_) _1,2_ + (βsA_f_) _1,2_ + (βI) _1,2_] | *Infanticidal takeovers:*  I_1_[(βA_m_) + (βA_f_) + (βsA_m_)] _2_ | 0 | I_1_Δ[(β_v_A_m_) + (β_v_A_f_) + (β_v_sA_m_) + (β_v_sA_f_)+ (β_v_I)]_2_ |
| UM | 0 | I[(βA_m_)_1,2_ + (βA_f_)_1,2_ + (βsA_m_) _1,2_ + (βsA_f_)_1,2_ + (βI) _1,2_] | *Infanticidal takeovers:*  I_1_[(βA_m_) + (βA_f_) + (βsA_m_)] _2_ | 0 | I_1_Δ[(β_v_A_m_) + (β_v_A_f_) + (β_v_sA_m_) + (β_v_sA_f_)+ (β_v_I)]_2_ |
| MO | 0 | I[(βA_m_)_1,2_ + (βA_f_) _1,2_ + (βI) _1,2_] | 0 | 0 | I_1_Δ[(β_v_A_m_) + (β_v_A_f_) + (β_v_I)]_2_ |
| SO  *Infants still in the natal group* | 0 |  | 0 | 0 | I_1_Δ[(β_v_A_m_) + (β_v_A_f_) + (β_v_I)]_2_ |

##### 7. Subdult Female Intergroup Transmission Possibilities

|  | STI | Fecal Oral | Aggression/Grooming | Aerosol | Vector |
| --- | --- | --- | --- | --- | --- |
| FF  *(Bonobos)* | ^a,b^εsA_f_) [(βA_m_) + (βA_f_) + (βsA_m_) + (βsA_f_) + (βI)]_2_  &  (isA_f_) [(βA_m_) + (βA_f_) + (βsA_m_) + (βsA_f_) + (βI)]_1_ | ^a,b^ (εsA_f_) [(βA_m_) + (βA_f_) + (βsA_m_) + (βsA_f_) + (βI)]_2_  &  (isA_f_) [(βA_m_) + (βA_f_) + (βsA_m_) + (βsA_f_) + (βI)]_1_ | *Emigrating and immigrating subadult females having fights*:  (εsA_f_) β_FIGHT_ [(A_m_) + (A_f_) + (sA_m_) + (sA_f_)]_2_  &  (isA_f_)β_FIGHT_ [ (A_m_) + (A_f_) + (sA_m_) + (sA_f_)]_1_ | ^a,b^(εsA_f_) [(βA_m_) + (βA_f_) + (βsA_m_) + (βsA_f_) + (βI)]_2_  &  (isA_f_) [(βA_m_) + (βA_f_) + (βsA_m_) + (βsA_f_) + (βI)]_1_ | sA_f1_Δ [(β_v_ A_m_) + (β_v_A_f_) + (β_v_I)]_2_ |
| MM | ^a,b^(εsA_f_) [(βA_m_) + (βA_f_) + (βsA_m_) + (βsA_f_) + (βI)]_2_  &  (isA_f_) [(βA_m_) + (βA_f_) + (βsA_m_) + (βsA_f_) + (βI)]_1_ | ^a,b^(εsA_f_) [(βA_m_) + (βA_f_) + (βsA_m_) + (βsA_f_) + (βI)]_2_  &  (isA_f_) [(βA_m_) + (βA_f_) + (βsA_m_) + (βsA_f_) + (βI)]_1_ | *Emigrating and immigrating subadult females having fights*:  (εsA_f_) [β_FIGHT_ (A_m_) + (A_f_) + (sA_m_) + (sA_f_)]_2_  &  (isA_f_) [β_FIGHT_ (A_m_) + (A_f_) + (sA_m_) + (sA_f_)]_1_ | ^a,b^(εsA_f_) [(βA_m_) + (βA_f_) + (βsA_m_) + (βsA_f_) + (βI)]_2_  &  (isA_f_) [(βA_m_) + (βA_f_) + (βsA_m_) + (βsA_f_) + (βI)]_1_ | sA_f1_Δ[(β_v_A_m_) + (β_v_A_f_) + (β_v_I)]_2_ |
| UM | ^a,b^(εsA_f_) [(βA_m_) + (βA_f_) + (βsA_m_) + (βsA_f_) + (βI)]_2_  &  (isA_f_) [(βA_m_) + (βA_f_) + (βsA_m_) + (βsA_f_) + (βI)]_1_ | ^a,b^(εsA_f_) [(βA_m_) + (βA_f_) + (βsA_m_) + (βsA_f_) + (βI)]_2_  &  (isA_f_) [(βA_m_) + (βA_f_) + (βsA_m_) + (βsA_f_) + (βI)]_1_ | *Emigrating and immigrating subadult females having fights*:  (εsA_f_) [β_FIGHT_ (A_m_) + (A_f_) + (sA_m_) + (sA_f_)]_2_  &  (isA_f_) [β_FIGHT_ (A_m_) + (A_f_) + (sA_m_) + (sA_f_)]_1_ | ^a,b^(εsA_f_) [(βA_m_) + (βA_f_) + (βsA_m_) + (βsA_f_) + (βI)]_2_  &  (isA_f_) [(βA_m_) + (βA_f_) + (βsA_m_) + (βsA_f_) + (βI)]_1_ | sA_f1_Δ[(β_v_A_m_) + (β_v_A_f_) + (β_v_I)]_2_ |
| MO | 0 | 0 | 0 | 0 | 0 |
| SO | 0 | 0 | 0 | 0 | 0 |

*^a^When subadult females are the age class that leaves the natal group*

*^b^Emigrating and immigrating subadult females having effects on groups 1 and 2***8. Subdult Male Intergroup Transmission Possibilities**

|  | STI | Fecal Oral | Aggression/Grooming | Aerosol | Vector |
| --- | --- | --- | --- | --- | --- |
| FF  *(Bonobos)* | ^a,b^ (εsA_m_) [(βA_m_) + (βA_f_) + (βsA_m_) + (βsA_f_) + (βI)]_2_  &  (iA_m_) [(βA_m_) + (βA_f_) + (βsA_m_) + (βsA_f_) + (βI)] | ^a,b^ (εsA_m_) [(βA_m_) + (βA_f_) + (βsA_m_) + (βsA_f_) + (βI)]_2_  &  (iA_m_) [(βA_m_) + (βA_f_) + (βsA_m_) + (βsA_f_) + (βI)] | ^a,b^ (εsA_m_ + iA_m_) [β_FIGHT_ (A_m_)_1,2_ + (A_f_)_1,2_ + (sA_m_) _1,2_ + (sA_f_) _1,2_] | ^a,b^ (εsA_m_) [(βA_m_) + (βA_f_) + (βsA_m_) + (βsA_f_) + (βI)]_2_  &  (iA_m_) [(βA_m_) + (βA_f_) + (βsA_m_) + (βsA_f_) + (βI)] | sA_m1_Δ[(β_v_A_m_) + (β_v_A_f_) + (β_v_sA_m_) + (β_v_sA_f_)+ (β_v_I)]_2_ |
| MM | ^a,b^ (εsA_m_) [(βA_m_) + (βA_f_) + (βsA_m_) + (βsA_f_) + (βI)]_2_  &  (iA_m_) [(βA_m_) + (βA_f_) + (βsA_m_) + (βsA_f_) + (βI)] | ^a,b^ (εsA_m_) [(βA_m_) + (βA_f_) + (βsA_m_) + (βsA_f_) + (βI)]_2_  &  (iA_m_) [(βA_m_) + (βA_f_) + (βsA_m_) + (βsA_f_) + (βI)] | ^a,b^ (εsA_m_ + iA_m_) [β_FIGHT_ (βA_m_)_1,2_ + (βA_f_)_1,2_ + (βsA_m_) _1,2_ + (βsA_f_) _1,2_ + (βI) _1,2_] | ^a,b^ (εsA_m_) [(βA_m_) + (βA_f_) + (βsA_m_) + (βsA_f_) + (βI)]_2_  &  (iA_m_) [(βA_m_) + (βA_f_) + (βsA_m_) + (βsA_f_) + (βI)] | sA_m1_Δ[(β_v_A_m_) + (β_v_A_f_) + (β_v_sA_m_) + (β_v_sA_f_)+ (β_v_I)]_2_ |
| UM | (εsA_m_) [(βA_m_) + (βA_f_) + (βsA_m_) + (βsA_f_) + (βI)]_2_  &  (iA_m_) [(βA_m_) + (βA_f_) + (βsA_m_) + (βsA_f_) + (βI)] | (εsA_m_) [(βA_m_) + (βA_f_) + (βsA_m_) + (βsA_f_) + (βI)]_2_  &  (iA_m_) [(βA_m_) +  (βA_f_) +(βsA_m_) + (βsA_f_) +(βI)] | (εsA_m_ + iA_m_) [β_FIGHT_ (βA_m_)_1,2_ + (βA_f_)_1,2_ + (βsA_m_) _1,2_ + (βsA_f_) _1,2_ + (βI) _1,2_] | (εsA_m_) [(βA_m_) + (βA_f_) + (βsA_m_) + (βsA_f_) + (βI)]_2_  &  (iA_m_) [(βA_m_) + (βA_f_) + (βsA_m_) + (βsA_f_) + (βI)] | sA_m1_Δ[(β_v_A_m_) + (β_v_A_f_) + (β_v_sA_m_) + (β_v_sA_f_)+ (β_v_I)]_2_ |
| MO | 0 | 0 | 0 | 0 | 0 |
| SO | 0 | 0 | 0 | 0 | 0 |

*^a^ When subadult males are the age class that leaves the natal group*

*^b^ Emigrating and immigrating subadult males having effects on groups 1 and 2***9. Adult Female Intergroup Transmission Possibilities**

|  | STI | Fecal Oral | Aggression/Grooming | Aerosol | Vector |
| --- | --- | --- | --- | --- | --- |
| FF  *(Bonobos)* | 0 | *Rate at which adult female ranges overlap other groups:*  A_f_[(βA_m_) + (βA_f_) + (βsA_m_) + (βsA_f_) + (βI)]_2_ | *Territorial and infant defense:*  A_f_ β_FIGHT_ [ (A_m_)_2_ + (A_f_)_2_ + (sA_m_)_2_ + (sA_f_)_2_] | *Rate at which adult female ranges overlap other groups:*  A_f_[(βA_m_) + (βA_f_) + (βsA_m_) + (βsA_f_) + (βI)]_2_ | A_f1_Δ[(β_v_A_m_) + (β_v_A_f_) + (β_v_sA_m_) + (β_v_sA_f_)+ (β_v_I)]_2_ |
| MM | 0 | *Rate at which adult female ranges overlap other groups:*  A_f_[(βA_m_) + (βA_f_) + (βsA_m_) + (βsA_f_) + (βI)]_2_ | *Territorial and infant defense:*  A_f_ β_FIGHT_ [(A_m_)_2_ + (A_f_)_2_ + (sA_m_)_2_ + (sA_f_)_2_] | *Rate at which adult female ranges overlap other groups:*  A_f_[(βA_m_) + (βA_f_) + (βsA_m_) + (βsA_f_) + (βI)]_2_ | A_f1_Δ[(β_v_A_m_) + (β_v_A_f_) + (β_v_sA_m_) + (β_v_sA_f_)+ (β_v_I)]_2_ |
| UM | 0 | *Rate at which adult female ranges overlap other groups:*  A_f_[(βA_m_) + (βA_f_) + (βsA_m_) + (βsA_f_) + (βI)]_2_ | *Territorial and infant defense:*  A_f_ β_FIGHT_ [(A_m_)_2_ + (A_f_)_2_ + (sA_m_)_2_ + (sA_f_)_2_] | *Rate at which adult female ranges overlap other groups:*  A_f_[(βA_m_) + (βA_f_) + (βsA_m_) + (βsA_f_) + (βI)]_2_ | A_f1_Δ[(β_v_A_m_) + (β_v_A_f_) + (β_v_sA_m_) + (β_v_sA_f_)+ (β_v_I)]_2_ |
| MO | 0^a^ | *Rate at which adult female joins with new partners:* A_f_[(βA_m_)]_2_ | *Territorial and infant defense:*  A_f_ β_FIGHT_ [(A_m_)_2_ + (A_f_)_2_] | *Rate at which adult female joins with new partners:* A_f_[(βA_m_)]_2_ | A_f1_Δ[(β_v_A_m_) + (β_v_A_f_) + (β_v_I)]_2_ |
| SO | 0^a^ | *Rate at which adult female joins with new partners:* A_f_[(βA_m_)]_2_ | *Territorial and infant defense:*  A_f_ β_FIGHT_ [(A_m_)_2_ + (A_f_)_2_] | *Rate at which adult female joins with new partners:* A_f_[(βA_m_)]_2_ | A_f1_Δ[(β_v_A_m_) + (β_v_A_f_) + (β_v_I)]_2_ |

^a^ *Except for serial monogamy* **10. Adult Male Intergroup Transmission Possibilities**

|  | STI | Fecal Oral | Aggression/Grooming | Aerosol | Vector |
| --- | --- | --- | --- | --- | --- |
| FF  *(Bonobos)* | 0 | *Rate at which adult male ranges overlap other groups:* A_m_[(βA_m_)_1,2_ + (βA_f_)_1,2_ + (βsA_m_)_1,2_ + (βsA_f_)_1,2_ + (βI)_1,2_] | *Territorial defense:*  A_m_ β_FIGHT_ [(A_m_)_2_ + (A_f_)_2_ + (sA_m_)_2_ + (sA_f_)_2_] | *Rate at which adult male ranges overlap other groups:* A_m_[(βA_m_)_1,2_ + (βA_f_)_1,2_ + (βsA_m_)_1,2_ + (βsA_f_)_1,2_ + (βI)_1,2_] | A_m1_Δ[(β_v_A_m_) + (β_v_A_f_) + (β_v_sA_m_) + (β_v_sA_f_)+ (β_v_I)]_2_ |
| MM | 0 | *Rate at which adult male ranges overlap other groups:* A_m_[(βA_m_)_1,2_ + (βA_f_)_1,2_ + (βsA_m_)_1,2_ + (βsA_f_)_1,2_ + (βI)_1,2_] | *Territorial defense:*  A_m_ β_FIGHT_ [(A_m_)_2_ + (A_f_)_2_ + (sA_m_)_2_ + (sA_f_)_2_] | *Rate at which adult male ranges overlap other groups:* A_m_[(βA_m_)_1,2_ + (βA_f_)_1,2_ + (βsA_m_)_1,2_ + (βsA_f_)_1,2_ + (βI)_1,2_] | A_m1_Δ[(β_v_A_m_) + (β_v_A_f_) + (β_v_sA_m_) + (β_v_sA_f_)+ (β_v_I)]_2_ |
| UM | 0 | *Rate at which adult male ranges overlap other groups:* A_m_[(βA_m_)_1,2_ + (βA_f_)_1,2_ + (βsA_m_)_1,2_ + (βsA_f_)_1,2_ + (βI)_1,2_] | *Territorial defense:*  A_m_ β_FIGHT_ [(A_m_)_2_ + (A_f_)_2_ + (sA_m_)_2_ + (A_f_)_2_] | *Rate at which adult male ranges overlap other groups:* A_m_[(βA_m_)_1,2_ + (βA_f_)_1,2_ + (βsA_m_)_1,2_ + (βsA_f_)_1,2_ + (βI)_1,2_] | A_m1_Δ[(β_v_A_m_) + (β_v_A_f_) + (β_v_sA_m_) + (β_v_sA_f_)+ (β_v_I)]_2_ |
| MO | 0^a^ | *Rate at which adult male joins with new partners:* A_m_[(βA_m_)_1,2_ + (βA_f_)_1,2_ + (βI)_1,2_] | *Territorial defense:*  A_m_ β_FIGHT_ [(A_m_)_2_ + (A_f_)_2_] | *Rate at which adult male ranges overlap other groups:* A_m_[(βA_m_)_1,2_ + (βA_f_)_1,2_ + (βI)_1,2_] | A_m1_Δ[(β_v_A_m_) + (β_v_A_f_) + (β_v_I)]_2_ |
| SO | ^b^A_m_(βAf)_n_ | *Rate at which adult male joins with new partners:*  A_m_[(βA_m_)_1,2_ + (βA_f_)_1,2_ + (βI)_1,2_] | *Territorial defense:*  A_m_ β_FIGHT_ [(A_m_)_2_ + (A_f_)_2_] | *Rate at which adult male ranges overlap other groups:* A_m_[(βA_m_)_1,2_ + (βA_f_)_1,2_ + (βI)_1,2_] | A_m1_Δ[(β_v_A_m_) + (β_v_A_f_) + (β_v_I)]_2_ |

*^a^ Except for serial monogamy*

*^b^ Serial sexual acts in n different groups*
