# Supplementary material for: Interactions between Social Structure, Demography, and Transmission Determine Disease Persistence in Primates
Source: PLoS One. 2013 Oct 18;8(10):e76863. doi: 10.1371/journal.pone.0076863 (PMC3800049; doi:10.1371/journal.pone.0076863)
Supplement: Table S2 — Life-history parameters for Small, Medium and Large (S, M, L) primates. (DOC) [file pone.0076863.s003.doc]

**Table S2**

| **Life history trait** | **Small** | **Medium** | **Large** |
| --- | --- | --- | --- |
| *Infant-juvenile stage (years)* | 0.14 | 3 | 6 |
| *Age of Sexual maturity (Male/Female) (years)* | 1.6/2 | 5/6 | 10/12 |
| *Natural lifespan (years)* | 4 | 15 | 30 |
| *Birthrate (per year)* | 1.15^a^ | 0.303 | 0.20 |
| *Population growth rate (per year)* | 1.016 | 1.016 | 1.016 |
| *Generation time, T (years)* | 6.33 | 20.07 | 36.75 |

^a^ occasional twinning

1. Ballenger L (2001) Galago senegalensis. Animal Diversity Web.

2. Hakeem A, Sandoval R, Jones M, Allman J (1996) Brain and life span in primates. In: Birren J, Schaie KW, editors. Handbook of the Psychology of Aging. 4th ed. San Diego: Academic Press. pp. 78–104.

3. Cawthon Lang KA (2005) Primate Factsheets: Gorilla (Gorilla) Taxonomy, Morphology, & Ecology
