# Supplementary material for: Interactions between Social Structure, Demography, and Transmission Determine Disease Persistence in Primates
Source: PLoS One. 2013 Oct 18;8(10):e76863. doi: 10.1371/journal.pone.0076863 (PMC3800049; doi:10.1371/journal.pone.0076863)
Supplement: Discussion S1 — From theory to reality: how can we establish and understand contact rates for these transmission modes? (DOCX) [file pone.0076863.s005.docx]

**Supplementary Discussion S1:** From theory to reality: how can we establish and understand contact rates for these transmission modes?

**Sexually Transmitted Infections (STIs)**

Rates at which STIs may be transmitted can best be quantified in terms of numbers of sexual encounters. Obtaining this information empirically is very complicated. For females, the information can be derived from analyses of the age of sexual maturity, age of first birth, interbirth interval, oestrus period and frequency, average life-span, and social system. Direct field observation of male behavior within groups while the females are in oestrus may be the most reliable field estimate of sexual encounter rate. Deriving possible opportunities from female life history characteristics, combined with male life-history for sexual maturity, length of position in a hierarchy, or as α-male, and life span can also be useful. In terms of sexual encounters as a mode of disease transmission, one ought to take into account all the behaviors surrounding actual mating, such as grooming and inspection and aggression. It is possible for a sexual encounter to provide a means for aerosol, aggressive and vector transmission, regardless of copulation success.

The number of infants infected with an STI in a population can only grow if infected mothers vertically transmit an STI to that infant, and the infant neither dies of the disease, nor clears the pathogen from its system before it is old enough to transmit it. This should select for a pathogen with high transmissibility, low morbidity, and long duration of infectivity, such as characterizes the human Herpes I and II STIs. Theoretically, this is a violation of the fundamental trade-off in pathogen life histories (between transmissibility and pathogenicity) [[1](#_ENREF_1),[2](#_ENREF_2)], which argues for strong selection on pathogen adaptation rather than social systems in primates.

In a MO or SO social system, where the infants remain with the family group until maturity, the latent period of the pathogen would have to exceed this growing time. Loehle [[3](#_ENREF_3)] points out that for a monogamously grouped population, migrating males are usually virgins, and can’t carry an STI to a new group unless they have acquired it through vertical transmission.

As social systems become more complex, the routes of transmission within groups for STIs are increased. Thus a species with a small body size and a simple SO or MO system may be afforded a lower level of risk from novel STIs. The contact network in our models for MM and FF are described as for bonobos (*Pan paniscus*), whose interesting sexual habits would make STI transmission most likely. We would thus expect the overall infection rate to increase rapidly in this system, due to high contact frequency.

For larger primates, there is more opportunity for infectious contacts than for smaller primates, simply due to accumulation over a prolonged lifetime, regardless of social system. Thus the level of infection can build up in the population, similarly to the phenomenon of the ratcheting of prevalence of chronic viral STIs with age in humans [[4](#_ENREF_4),[5](#_ENREF_5)]. Therefore, we suggest that in the case of a species such as the siamang *(Symphalangus syndactylus)*, whose social system is an exception for its body size, it is unlikely that monogamy evolved as an escape from STIs. The converse may also be true: the evolution of more complex social systems creates opportunities for a greater diversity of pathogens, while species that are constrained to territorial monogamy (perhaps due to ecological constraints) should be hosts only to a predominance of STI’s.

Social stratification may influence prevalence, as dominance hierarchies introduce differential mating opportunities. Alpha males may gain reproductive fitness benefits by having the highest number of copulations, but they may also have a higher rate of STI acquisition. Under semi-natural conditions it was shown that alpha or dominant males of Olive baboons (*Papio anubis),* Rhesus macaques (*Macaca mulatta)* and Chacma baboons (*Papio ursinus)* gained 50-100% of all copulations [[6](#_ENREF_6),[7](#_ENREF_7)]. It was also noted that the alpha males tended to gain first copulations during female ovulation periods. Hausfater and Watson [[8](#_ENREF_8)] noticed that sexually cycling females passed more parasite eggs than anestrous females. This suggests that alpha males, while furthering their reproductive success may be simultaneously more vulnerable to sexually transmitted parasites. High ranking or dominant females may also face trade-offs: in the lab, dominant females of the Common marmoset (*Callithrix jacchus*) and the Pigtail macaque (*Macaca nemestrina*) gained up to 100% of all copulations [[6](#_ENREF_6)]. Higher ranked females may solicit more sexual encounters to procure parental care for their offspring, and prevent subdominant females from reproducing, but at a price. Concealed ovulation has evolved in many non-monogamous systems, which correlates with a higher level of non-reproductive copulations [[9](#_ENREF_9)], perhaps representing a trade-off between increased paternal care for infants through confused paternity, and the risk of acquiring STIs. Loehle [[3](#_ENREF_3)] even suggests that monogamy may have evolved from polygynous behaviour through pressure to avoid STIs. However, we again emphasize that the rates of pathogen and social evolution are so mismatched that this is unlikely.

Larger, longer-lived primates in more complex social systems will have more complex social development. This corresponds to a later age of maturity and first age of breeding [[10](#_ENREF_10)], so an STI would have to adapt or fade out. Longer infancy means that for an STI to establish from initial vertical transmission, the latent period and survival of the individual host would have to extend at least until sexual maturity, or the disease could not persist past vertical transmission.

Our model results showed that whereas STI transmission appears to increase as social complexity increases, the underlying demography governs whether the disease can persist in the simpler social systems. In SO or MO systems, the age of sexual maturity may tend to be earlier in absolute time than in a multimale (MM) or fission-fusion (FF) social system, this is primarily a consequence of the correlation between body size and social system. But the age at which larger primates in a MM or FF social system first engage in sexual encounters could be low enough to compensate for this difference. Thus an STI pathogen could retain similar virulence and latency across varying social systems.

The contrast between philopatric and dispersal strategies of subadults greatly impacts STI spread through a primate population. Female philopatry essentially creates a dead-end for intergroup transmission, so long as within group mating fidelity exists. However, for smaller MO or only seasonally monogamous primates, whose wean-and-go strategy of dispersal creates a periodically connected and dynamic network, an STI could persist and spread provided the host remained both alive and sufficiently mobile to allow transmission to occur.

# **Fecal-Oral Transmission**

For parasitic diseases of primates with a free-living stage in their life cycle (nematodes and helminthes, e.g.), the force of infection is dependent upon a combination of encounter rate and infectious potential of the free-living stage. In our contact matrices, we assumed that all members of the social group encountered each other’s feces at the same rate, thus intra-group transmission scales with group size. This reduces to the fully-mixed intra-group scenario, so within group infections will be largely driven by demographic turnover and by movement across the metapopulation. We assumed discrete groups, meaning that intergroup infection occurs only via dispersal of infected individuals.

Loehle [[3](#_ENREF_3)] and Freeland [[11](#_ENREF_11)] both pointed out that increased numbers of pathogen types are associated with increased group sizes, so qualitatively, SO and MO groups would again be implicitly at lower risk. However, many monogamous species sleep in large aggregate groups [[12](#_ENREF_12),[13](#_ENREF_13)], which may enhance rates of FO transmission.

Fecally transmitted parasites may also play a role in the rate at which primates change their home ranges or sleeping areas. Baboon troops relocate their sleeping area when it becomes dirty with feces and appear to return at a rate roughly corresponding to the limit of survival of free-living nematode larval stages [[8](#_ENREF_8)]. As baboon troops sleep in groups of up to 100 [[14](#_ENREF_14)], the rate of larval stage exposure could become dangerous relatively quickly, suggesting that relocation may be largely a parasite avoidance strategy. In addition, it is thought that many species are meticulous about defecation, carefully using an area outside the sleeping and eating area within the range for feces. As pointed out by Nunn and Altizer [[15](#_ENREF_15)], this has only been explicitly observed for a few species [[16](#_ENREF_16),[17](#_ENREF_17)]. Mangabeys, (*Cercocebus albigena)* were reported to contaminate even the vegetation where they walk and eat, although Freeland [[18](#_ENREF_18)] noted that they changed their ranges less frequently with rainfall, suggesting they may rely on rain to wash away feces.

Empirical studies suggest that parasite burdens change with dominance ranking. Intuitively one might think that lower status in a hierarchy would correlate to higher parasite burdens, as lower status may be stressful. However, studies have shown that higher-ranking individuals had more intestinal parasites [[8](#_ENREF_8)], which may be a result of stress-induced weakening of immune defenses as the individual rises in the dominance hierarchy, as suggested by Sapolsky [[19](#_ENREF_19)]. Ultimately, this type of individual could prove to be of fundamental importance to the disease network at the population level, as the individual may attempt to join a new group and spread the parasites immediately after being deposed and ejected from the social group while at the peak of both rank and stress.

To more accurately describe fecal-oral transmission for an empirical system, we would need a spatially explicit model of the system, requiring information on overlap between multiple groups’ home ranges, in addition to defecation habits and parasite survival and virulence. With increasing sophistication in data collection, such as high-resolution GPS collars [[20](#_ENREF_20)], and wider availability of agent-based modeling software, these data-hungry models will become more plausible in the near future.

#### *Agonistic Interactions*

Aggressive interactions, including dominance struggles, attempts at infanticide, and agnostic encounters of emigrants, probably contribute to intra- and intergroup rates of disease transmission. In our behavioral interaction matrices (Supplementary Table 1) we assumed that MO and SO agonistic encounters only occur for adults, resulting from territory defense or intergroup invasions.

In UM and MM groups, the rate of intragroup aggression can be quite high, as reports of infanticide [[21](#_ENREF_21),[22](#_ENREF_22),[23](#_ENREF_23),[24](#_ENREF_24),[25](#_ENREF_25),[26](#_ENREF_26),[27](#_ENREF_27)] and male dominance fights [[25](#_ENREF_25),[26](#_ENREF_26),[28](#_ENREF_28)] are common. Loehle [[3](#_ENREF_3)] suggests that the complex facial expressions, gestures and vocalisations that typify many intergroup fights may have evolved to limit disease transmission. During migration, subadults encounter agonistic responses that may affect disease transmission in two ways. Firstly, the entering individual may have to fight to join the group and suffer from wounds that consequently become infected (although not by transmissible agents), causing susceptibility and immune weakening. Secondly, the stress induced by migration may increase the individual’s susceptibility to disease, increasing the probability that the individual is infectious, making dispersing individuals a risky prospect. The tradeoff between dispersal and disease risk was highlighted by Freeland [[11](#_ENREF_11)], who suggested that females may have greater success staying in their natal groups, due to increased disease risks of trying to join another. Behavioral data collected on agonistic interactions could be used to establish detailed forms of the contact matrices.

As a contrasting mode of contact, grooming may reduce the rate at which primates suffer from ectoparasites such as mites, lice and ticks. As this plays an important role in signaling rank in social behavior, the rate of ectoparasite removal is probably quite epidemiologically significant in primates with complex dominance hierarchies. Grooming matrices are quite a common form of behavioral observation in the primate literature [[29](#_ENREF_29)], and could be used to quantify contact rates for specific populations. Ectoparasite removal may reduce vector transmission of disease, but might also result in direct contact transmission due to close proximity.

In MO and SO, grooming rates are unlikely correlated to social status so ectoparasite prevalence may be consistent across age/stage classes. However, many MO groups are seen interacting on a population level and although intergroup grooming is unlikely it may occur. In hierarchically dominated social systems (MM and FF) in which grooming is used for favours, the effect of ectoparasite removal is likely to vary across the age/gender categories. The benefits of reducing ectoparasites will be most pronounced for those who receive the most grooming. Concomitantly, the close proximity required from grooming may lead to increased rates of direct transmission of a range of other pathogens.

##### Direct Transmission

The transmission of aerosolized viral or bacterial infection is strongly governed by the pathogen itself. The latent period, infectious period and mortality induced by the disease play key roles in its dynamics and persistence in the host population.

Our models describe an increase of direct contacts as social system complexity increases. Similarly to the FO route, in AERO there are no age-specific differences in our WAIFW matrices. In reality, age-specific behaviors such as infant play and grooming session duration will determine mixing levels within the group, while at the metapopulation level, age-related movement behavior such as migration and territorial defense will modulate and determine transmission rates. Data collected on activity budgets and ranging could be used to parameterize these components.

In a spatially implicit metapopulation such as the one modeled here, a pathogen would be most successful in causing epidemics if it had a long latent and infectious period and did not disable individuals in one group to the extent that they could not overlap with individuals of other groups. In a spatially explicit metapopulation model, such as a lattice or grid based arrangement, provided a threshold level of contacts exists that allows the chain of infection to persist, a randomly seeded infection should create a radiating epidemic from an initial epicenter in an arena of evenly spaced groups. But the true geographical configuration of groups could be important to determining the dynamics of spread and persistence. For example, recent concerns about respiratory viruses transmitted to research populations of chimpanzees by researchers themselves [[30](#_ENREF_30)] resemble spillover events of infection at the edge of a range, rather than a random occurrence within a metapopulation. The actual spatial structure and group locations will be important if control measures for disease spread, such as vaccinations or fencing, are under consideration. In particular it will likely prove easier to break the chain of transmission and halt the spread of infection if the social groups are organized as an almost linear array than of they were arranged as a tessellation across a plain

***Vector transmission***

In our models, VEC is reduced to a function of group size. The WAIFW matrix is the fully saturated, mass-action transmission probability. As pointed out above, the reduction of ectoparasitic vectors via grooming may be one benefit of group living, but only for those receiving the grooming favors. Similarly, while grooming reduces the abundance of ticks and fleas, the clouds of CO2 produced by a group of individuals will increase their attraction to mosquitos and other flying insect vectors that are less likely to be reduced in abundance by grooming.

Socioecological models suggest that one benefit of increasing group size is to ‘dilute’ negative events such as predation, and, one might assume, insect bites. This within group ‘dilution effect’ operates in ways that are directly analogous to Hamilton’s ‘selfish herd’ effect [[31](#_ENREF_31)]. However, sleeping group size was shown to positively correlate with mosquito biting rate in a study of malaria in Amazonian primates [[13](#_ENREF_13)].The ability of mosquitoes and ticks to find and take blood meals from warm-blooded mammals, particularly infected ones [[32](#_ENREF_32),[33](#_ENREF_33),[34](#_ENREF_34)] may simply overwhelm the potential dilution effects where vectors exist in high densities. Rather than protection conferred by being part of a larger group, there may be a selective effect towards smaller group sizes to avoid increased vectoral capacity of a disease.

**Supplemental Discussion References**

1. van Baalen M, Sabelis MW (1995) The dynamics of multiple infection and the evolution of virulence. Am Nat 146: 881-910.

2. Frank SA (1996) Models of Parasite Virulence. The Quarterly Review of Biology 71: 37.

3. Loehle C (1995) Social barriers to pathogen transmission in wild animal populations. Ecology 76: 326-335.

4. Brugha R, Keersmaekers K, Renton A, Meheus A (1997) Genital herpes infection: a review. Int J Epidemiol 26: 698-709.

5. Smith JS, Robinson NJ (2002) Age-Specific Prevalence of Infection with Herpes Simplex Virus Types 2 and 1: A Global Review. The Journal of Infectious Diseases 186: S3-S28.

6. Dewsbury DA (1982) Dominance rank, copulatory behavior, and differential reproduction. The Quarterly Review of Biology 57: 159.

7. Beehner JC, Onderdonk DA, Alberts SC, Altmann J (2006) The ecology of conception and pregnancy failure in wild baboons. Behav Ecol 17: 741-750.

8. Hausfater G, Watson DF (1976) Social and reproductive correlates of parasite ova emissions by baboons. Nature 262: 689.

9. Sillen-Tullberg B, Moller AP (1993) The relationship between concealed ovulation and mating systems in anthropoid primates - a phylogenetic analysis. Am Nat 141: 1-25.

10. Charnov EL, Berigan D (1993) Why do female primates have such long lifespans and so few babies? or Life in the slow lane. Evol Anthropol: 191-194.

11. Freeland WJ (1976) Pathogens and the evolution of primate sociality. Biotropica 8: 12-24.

12. Ibanez AE (1986) Interanimal distance: spacing and social structure. In: Mitchell G, Erwin J, editors. Comparative primate biology, volume 2a: behavior, conservation and ecology. New York: Alan R. Liss, Inc. pp. 169-194.

13. Davies CR, Ayres JM, Dye C, Deane LM (1991) Malaria infection rate of Amazonian primates increases with body weight and group size. Funct Ecol 5: 662.

14. Strum SC, Western JD (1982) Variations in fecundity with age and environment in olive baboons (*Papio anubis*). Am J Primatol 3: 61-76.

15. Nunn CL, Altizer S (2006) Infectious diseases in primates: behavior, ecology, evolution. Oxford: Oxford University Press.

16. Henry RE, Winkler L (2001) Foraging, feeding and defecation site selection as a parasite avoidance strategy of *Alouatta palliata* in a dry tropical forest. Am J Phys Anthropol Suppl 32: 79.

17. Gilbert KA (1997) Red howling monkey use of specific defecation sites as a parasite avoidance strategy. Anim Behav 54: 451-455.

18. Freeland WJ (1980) Mangabey (*Cerocebus Albigena*) Movement Patterns in Relation to Food Availability and Fecal Contamination. Ecology 61: 1303.

19. Sapolsky RM (2005) The Influence of Social Hierarchy on Primate Health. Science 308: 648-652.

20. Crofoot MC, Gilby IC, Wilkelski MC, Kays RW (2008) Interaction location outweighs the competitive advantage of numerical superiority in *Cebus capucinus* intergroup contests. Proc Nat Acad Sci USA 105: 577-581.

21. Lycett JE, Henzi SP, Barrett L (1998) Maternal investment in mountain baboons and the hypothesis of reduced care. Behav Ecol Sociobiol 42: 49-56.

22. Crockett CM, Janson CH (2000) Infanticide in red howlers: female group size, male membership, and a possible link to folivory. In: van Schaik CP, Janson CH, editors. Infanticide by males and its implications. Cambridge: Cambridge University Press. pp. 75-98.

23. Janson CH, Goldsmith ML (1995) Predicting group-size in primates - foraging costs and predation risks. Behav Ecol 6: 326-336.

24. Hill RA, Dunbar RIM (1998) An evaluation of the roles of predation rate and predation risk as selective pressures on primate grouping behaviour. Behaviour 135: 411-430.

25. Kitchen DM, Horwich RH, James RA (2004) Subordinate male black howler monkey (*Alouatta pigra*) responses to loud calls: Experimental evidence for the effects of intra-group male relationships and age. Behaviour 141: 703-723.

26. Kitchen DM (2004) Alpha male black howler monkey responses to loud calls: effect of numeric odds, male companion behaviour and reproductive investment. Anim Behav 67: 125-139.

27. Knopff KH, Knopff ARA, Pavelka MSM (2004) Observed case of infanticide committed by a resident male central American black howler monkey (*Alouatta pigra*). Am J Primatol 63: 239-244.

28. Schaik CP, Noordwijk MA (1989) The special role of male *Cebus* monkeys in predation avoidance and its effect on group composition. Behav Ecol Sociobiol 24: 276.

29. Lehmann J, Korstjens AH, Dunbar RIM (2007) Group size, grooming and social cohesion in primates. Anim Behav 74: 1629.

30. Kondgen S, Kuhl H, N'Goran PK, Walsh PD, Schenk S, et al. (2008) Pandemic human viruses cause decline of endangered great apes. Curr Biol 18: 260-264.

31. Hamilton WD (1971) Geometry for the selfish herd. J Theor Biol: 295-311.

32. Turell MJ, Bailey CL, Rossi CA (1984) Increased Mosquito Feeding on Rift Valley Fever Virus-Infected Lambs. Am J Trop Med Hyg 33: 1232-1238.

33. Belan I, Bull CM (1991) Host Detection by Four Australian Tick Species. J Parasitol 77: 340.

34. Lacroix R, Mukabana WR, Gouagna LC, Koella JC (2005) Malaria Infection Increases Attractiveness of Humans to Mosquitoes. PLoS Biol 3: e298.
